# Resource Use Patterns in US Telehealth Services: Machine Learning and Clustering Analysis Across 4 Specialties

**DOI:** 10.2196/78030

**Published:** 2026-05-07

**Authors:** Aysenur Betul Cengil, Burak Eksioglu, Sandra Duni Eksioglu, Corey Hayes, Cari Bogulski, Mir Ali

**Affiliations:** 1 Department of Industrial Engineering University of Arkansas Fayetteville, AR United States; 2 Department of Industrial, Manufacturing and Systems Engineering Texas Tech University Lubbock, TX United States; 3 Institute for Digital Health and Innovation University of Arkansas for Medical Sciences Little Rock, AR United States; 4 Department of Biomedical Informatics University of Arkansas for Medical Sciences Little Rock, AR United States; 5 Center for Mental Healthcare and Outcomes Research Central Arkansas Veterans Healthcare System Little Rock, AR United States

**Keywords:** telehealth, resource use, machine learning, clustering, COVID-19 pandemic, deep neural networks

## Abstract

**Background:**

The expansion of telehealth services, particularly during the COVID-19 pandemic, has transformed health care delivery in the United States. Telehealth promises greater access and resource efficiency by reducing wait times and appointment lengths, especially in specialties like psychiatry, behavioral health, bariatrics, and sleep medicine. However, disparities exist in adoption based on demographics, geography, and socioeconomic status, raising concerns about equitable access and optimal resource use.

**Objective:**

This study aims to evaluate how telehealth impacts health care resource use across 4 specialties by examining 2 key metrics: patient-to-provider ratios and appointment durations. It seeks to understand how factors such as patient demographics, facility characteristics, and social determinants influence telehealth adoption and efficiency using a national dataset spanning from 2018 to 2023.

**Methods:**

We analyzed a deidentified dataset from Epic Cosmos, covering outpatient visits across 48 US states (2018-2023). After data preprocessing and feature engineering, we applied 3 machine learning (ML) models (random forest, extreme gradient boosting, and deep neural networks) to predict resource use. Using the model performing the best, feature importance was assessed using Shapley Additive Explanations values. We then used k-means clustering to group facilities into clusters per specialty. Comparative analyses were conducted to evaluate differences in use among clusters, during and after the pandemic.

**Results:**

Telehealth use peaked in 2020 and has remained above prepandemic levels since then. In 2018-2023, telehealth adoption reached 36.9% (4,543,021/12,311,710) in psychiatry, 23.9% (5,321,099/22,264,013) in behavioral health, 21.2% (924,333/4,360,061) in bariatrics, and 16.8% (851,803/5,070,256) in sleep medicine. Telehealth visits were consistently shorter than office visits (mean reduction 12.24 minutes; SD 3.33 minutes; *P*=.18), while patient-to-provider ratios varied significantly across specialties. Among ML models, extreme gradient boosting regression achieved the best performance (patient-to-provider ratios: *R*^2^=0.96-0.99; appointment durations: *R*^2^=0.61-0.69). Shapley Additive Explanations analysis identified visit type, telehealth use, facility size, rurality, and Social Vulnerability Index household vulnerability as the strongest predictors. Comparative analyses showed significant differences across clusters (all *P*<.05).

**Conclusions:**

Telehealth has become a sustainable component of health care, enhancing access and efficiency across both rural and urban areas. However, its impact varies across specialties and regions, highlighting the need for targeted strategies such as staffing support for vulnerable populations, infrastructure investments in rural facilities, and reimbursement models that reflect telehealth’s resource use. This study provides robust evidence from ML and clustering analyses, demonstrating how telehealth shapes resource use and offering actionable insights for equitable and sustainable integration.

## Introduction

The health care industry is undergoing a significant transformation due to rapid technological advancements, particularly the rise of telehealth [1]. Telehealth includes a range of remote health care services, bridging gaps between health care demand and supply by providing convenient access to consultations, remote monitoring, and timely interventions [2]. In the United States, telehealth adoption accelerated during the COVID-19 pandemic, highlighting its role in improving access to care [3].

Health care resources include medical personnel, equipment, and facilities required for service delivery [4]. For telehealth, resources also include digital platforms, communication devices, and technical support, while office visits rely on clinical spaces and on-site staff [5]. Resource use measures how efficiently these resources are managed, with studies indicating that telehealth can reduce wait times, increase visit rates, and minimize missed appointments [2,6-10], enhancing care continuity.

Telehealth services were provided by a diverse range of health care facilities. Some adopted telehealth for the first time in response to the pandemic, while others, which already had telehealth services in place, experienced increased demand over the past 4 years. Consequently, facilities had to allocate resources—such as doctors and nurses—across multiple types of visits, including office visits, telehealth appointments, and emergency calls. A study found that larger facilities delivered a higher proportion of their visits through telehealth compared to smaller facilities with fewer doctors and nurses [11]. This suggests that health care facilities vary in their resource use and staffing needs.

Identifying the factors that influence telehealth use and evaluating its impact on health care resources are essential for effective resource planning. Several studies indicate that factors such as technological infrastructure, patient acceptance, and broadband access determine the feasibility and effectiveness of implementing telehealth [12-14]. These studies also highlight variations in adoption across different health care specialties and patient demographics.

One recent study reported that telehealth services improved resource use, as telehealth visits were associated with shorter waiting times and appointment durations compared to office visits in certain specialties. Additionally, both waiting times and appointment durations for telehealth services decreased over the study period [15]. Another study identified disparities in telehealth use, consistent with findings from various data sources and periods [16]. It calls for further research to determine whether differences in telehealth service proportions are linked to hospital or community telehealth capacity and to understand why these service proportions vary across demographic characteristics. These findings highlight the importance of nationwide analyses to assess telehealth use comprehensively.

This study aims to identify factors influencing resource use in health care facilities in the United States and to compare patterns across different settings (telehealth and office visits) and time frames. We focus on 2 metrics, patient-to-provider ratio and appointment duration, as key indicators of how health care resources are allocated and managed. The patient-to-provider ratio reflects the distribution of patient load among providers, serving as an indicator of resource allocation within health care systems. Higher ratios may indicate overburdened providers and diminished quality of care, whereas lower ratios suggest more manageable workloads and potentially improved patient attention. Appointment duration captures how providers allocate time per visit, offering insight into time management and the depth of patient engagement. Together, these metrics provide a comprehensive view of health care resource use in both telehealth and office-based contexts. Using national data from 2018 to 2023, the study seeks to generate new insights into telehealth adoption and resource use, offering a broader perspective than previous research [17-24]. In doing so, it provides evidence to inform health care providers, policymakers, and other stakeholders in planning for sustainable telehealth integration.

Building on these objectives, we focus on 4 specialties, psychiatry, behavioral health, bariatrics, and sleep medicine, due to their relatively higher levels of telehealth use. Specifically, the study examines whether telehealth use and patient- and facility-related factors are associated with variations in patient-to-provider ratios and appointment durations. It also explores whether clusters of health care facilities demonstrate distinct patterns in telehealth adoption, patient-to-provider ratios, and appointment durations over time.

We hypothesize that (1) telehealth visits are associated with lower patient-to-provider ratios and shorter appointment durations than office visits, (2) patient- and facility-related factors significantly impact resource use across care settings, and (3) clusters of health care facilities demonstrate meaningful differences in telehealth adoption and performance metrics (patients per provider and appointment duration) over time.

## Methods

### Ethical Considerations

Data collection was approved by the institutional review board of the University of Arkansas for Medical Sciences (IRB 276339). It was determined as nonhuman subject research. The data were deidentified, and there was no interaction with individuals.

### Data Collection

Patient data for this study were sourced from Epic Cosmos (hereafter referred to as Cosmos), a robust platform offering a diverse and representative sample of patients across various demographics, including race, sex, age, geographic settings, and insurance types [25]. The dataset spanned patient visits from health care facilities across 48 US states and was fully deidentified to ensure confidentiality. Access to the dataset was facilitated through the Epic Systems Cosmos research platform via a Microsoft SQL Server database.

This study focused on telehealth (audiovisual) and office (face-to-face) visits in the United States between 2018 and 2023, capturing pre-, mid-, and postpandemic periods to evaluate the impact of the COVID-19 pandemic on telehealth adoption and resource use.

Our study used the following data tables from Cosmos:

- Encounter table: Details visit-related information, including encounter type (eg, office visits and telehealth), provider details, health care specialties, and appointment times.
- Patient table: Provides patient-related demographics and enables analysis of telehealth access across various populations (eg, older people and rural residents). It also includes the Centers for Disease Control and Prevention or the Agency for Toxic Substances and Disease Registry Social Vulnerability Index (SVI), offering insights into education, financial resources, and transportation needs.
- Insurance table: Contains coverage details for each visit, allowing identification of primary insurance providers.
- Hospital table: Includes data transfer start and end dates for each hospital, aiding in data cleaning processes.

### Data Preprocessing

#### Overview

Data preprocessing, including data cleaning and feature engineering, ensured and improved model performance [26]. This study focused on patient- and facility-related factors and on specialties with the highest rates of telehealth adoption to identify best practices for virtual care.

We processed visit-level data for these specialties using features derived from patient, encounter, insurance, and hospital tables. Recent studies frequently examined demographic factors, including age, sex, race, and rurality [20,27-30]. Age was a key variable, with research comparing use rates across age groups to understand adoption trends. Sex and racial disparities were also explored to identify inequities in telehealth access, while rurality was examined to evaluate telehealth’s role in addressing health care gaps in remote areas. Additionally, insurance coverage was a critical determinant, influencing telehealth access through policy and reimbursement mechanisms. These demographic insights informed strategies to enhance equitable access.

We also incorporated the SVI percentiles from the Centers for Disease Control and Prevention to assess the impact of social vulnerability on telehealth use [20,31]. The SVI ranked census tracts based on socioeconomic status, household characteristics, minority and language status, and housing type and transportation. The SVI metric aided in resource allocation for vulnerable populations, enhancing public health preparedness and response efforts [32].

#### Data Cleaning

**Figure 1:**
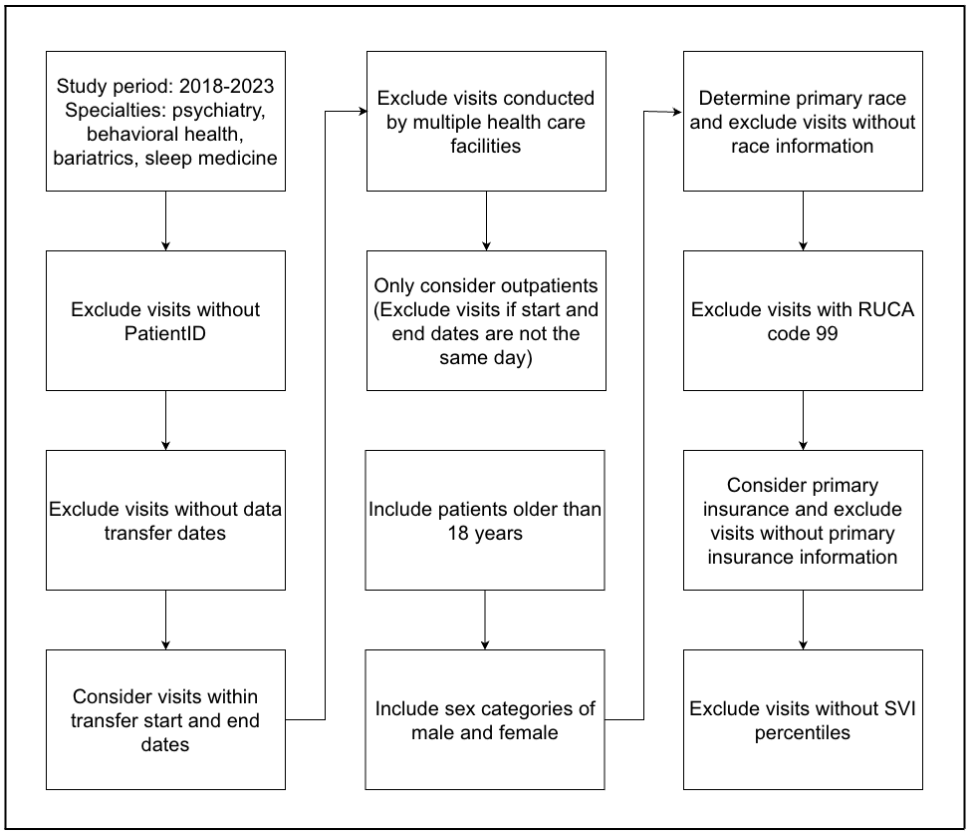
Flow diagram for data preprocessing steps. RUCA: rural-urban commuting area; SVI: Social Vulnerability Index.

The process used to define the subset of visits included in our study is illustrated in Figure 1. We excluded visits without a valid PatientID or transfer date to ensure data completeness. Because facilities periodically transfer electronic health record data, only visits within valid transfer start and end dates were included. We analyzed only same-day outpatient visits and focused on age, race, sex, rurality, insurance, and SVI percentiles. Preprocessing steps for these factors are shown in Figure 1.

Data were aggregated at the hospital level by month across 72 periods (2018-2023). Table 1 lists the variables, including temporal features (year, quarter, and month), to capture time trends. For demographics (race, sex, and insurance), we calculated the proportion of telehealth versus office visits; for age, rural-urban commuting area (RUCA), and SVI, we computed median age, average RUCA, and average SVI scores. To evaluate resource use, we calculated the patient-to-provider ratio and appointment duration for telehealth and office visits at each hospital and during each period. Appointment durations shorter than 10 minutes or longer than 100 minutes were excluded to reduce the influence of outliers and potential data entry errors. While such durations may occur in rare cases, they fall outside the typical range for standard provider-patient interactions and could skew the analysis. Finally, we scaled the training data before applying machine learning (ML) models [33].

**Table 1:** Variables and feature types.

Independent variables	Feature type
Year	Temporal
Quarter	Temporal
Month	Temporal
Age	Patient-related
RUCA^a^ code	Patient-related
Sex	Patient-related
Race	Patient-related
SVI^b^ household	Patient-related
SVI housing	Patient-related
SVI minority	Patient-related
SVI socioeconomic	Patient-related
Public insurance	Patient-related
Private insurance	Patient-related
Self-pay	Patient-related
Visit type	Patient-related
Number of visits	Facility-related
Number of providers	Facility-related
Telehealth use	Facility-related
Rurality	Facility-related
Total visits	Facility-related

^a^RUCA: rural-urban commuting area.

^b^SVI: Social Vulnerability Index.

### Model Development

#### Overview

**Figure 2:**
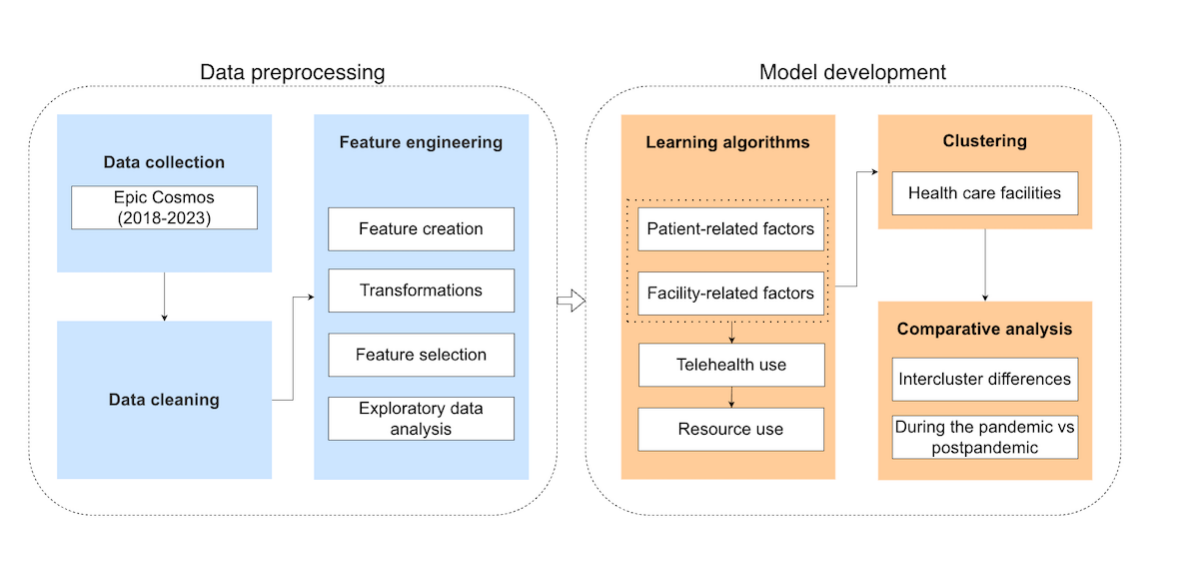
Flow diagram of the proposed methodology.

We conducted a comparative analysis of data from multiple health care facilities (2018-2023) to evaluate the impact of patient characteristics and health care settings on resource use and to identify key drivers. Given the large number of facilities, we used clustering to group similar facilities into meaningful profiles. This approach reduced complexity and highlighted common patterns across facility types, such as those with similar size, service mix, or adoption levels. By comparing these clusters, we provided insights that go beyond individual facilities and offered a clearer understanding of how telehealth influences resource use. In practice, this allowed facilities to benchmark their performance against comparable peers and consider alternative strategies. After clustering, we compared resource use between facility groups during and after the pandemic. Figure 2 illustrates the overall methodology, with details provided in the subsequent section.

#### Learning Algorithms

To examine the influence of patient- and facility-related factors on resource use, specifically the patient-to-provider ratio and appointment durations, we used nationwide temporal data and applied ML techniques. Prior work showed that ML improved performance with large health care datasets by uncovering links between demographics, clinical characteristics, and service types [34,35].

We evaluated 3 ML regression models: random forests, extreme gradient boosting (XGBoost), and deep neural networks (DNNs). These models were selected because they are widely used and complementary—random forests provide robustness and interpretability, XGBoost is highly efficient for structured tabular data, and DNNs are well-suited for capturing complex nonlinear relationships in large-scale datasets [36,37]. To optimize performance, we used Optuna, a hyperparameter tuning framework, to efficiently explore the parameter space and identify the best configuration for each specialty [38]. Model performance was assessed using *R*^2^, which measures how well predicted outcomes align with actual values and enables comparison across models and datasets [39]. To determine the relative importance of features on resource use outcomes, we used Shapley Additive Explanations (SHAP), which computes SHAP values to provide interpretable insights into feature contributions [40].

#### Clustering Model

After evaluating factors influencing resource use, we clustered health care facilities based on shared characteristics so that similar facilities can be analyzed collectively. The model included patient- and facility-related features and resource use metrics. To prepare the data for clustering, we aggregated it at the hospital level using the following methodology:

- Weighted averages: We calculated weighted averages using the number of visits as the weighting factor for the following patient-related factors: age, RUCA code, SVI household, SVI housing, SVI minority, SVI socioeconomic, race, sex, and payment methods (public insurance, private insurance, and self-pay).
- Patient-to-provider ratio: We calculated the weighted average of this ratio with the number of providers as the weighting factor.
- Telehealth use: We summed the total number of telehealth visits and overall visits for each hospital. The proportion of telehealth visits was then determined by dividing the number of telehealth visits by the total number of visits.
- Provider counts: We aggregated the total number of providers offering telehealth and office visits for each hospital.
- Rurality variable: This variable remained unchanged, as it was already defined at the hospital level.

The features initially considered in the clustering model are detailed in Textbox 1. We used *k*-means clustering, a widely used algorithm for partitioning datasets into distinct groups based on feature similarity [41]. First, we evaluated feature correlations and excluded those with a correlation coefficient equal to or higher than 0.70 [42]. Then, we further refined the clustering process by removing outliers using the isolation forest algorithm [43].

**Variables for the clustering model.**

**Independent variables**

- Age
- Rural-urban commuting area code
- Social Vulnerability Index (SVI) household
- SVI housing
- SVI minority
- SVI socioeconomic
- Race
- Sex
- Public insurance
- Private insurance
- Self-pay
- Appointment durations
- Rurality
- Total visits
- Number of telehealth visits
- Number of providers
- Patient-to-provider ratio
- Telehealth use

To determine the optimal number of clusters, we applied the elbow method [44]. The elbow point marked the balance between minimizing inertia and avoiding excessive clustering. Additionally, we considered cluster evaluation metrics, including the Davies-Bouldin Index, where lower values indicate better-defined clusters [45], and the Calinski-Harabasz Index, which favors higher values for compact and well-separated clusters [46]. Once the optimal number of clusters was identified for each specialty, we executed separate clustering models for each specialty. Finally, we assessed feature importance within the clustering model using the variance explanation method [47].

#### Comparative Analysis

After clustering health care facilities, we analyzed disparities in patient-to-provider ratios and appointment durations. Although we collected data for 3 time periods: pre–COVID-19 (2018-2019), during COVID-19 (2020-2021), and post–COVID-19 (2022-2023), the prepandemic years are excluded due to the limited telehealth adoption.

To investigate whether statistically significant differences exist in appointment durations or patient-to-provider ratios among the clusters of health care facilities, we applied 1-way ANOVA [48]. These analyses are conducted separately by specialty, visit type (telehealth and office visits), and time period (during and postpandemic). The hypotheses are defined as follows:

- H_0_: There are no statistically significant differences in patient-to-provider ratios or appointment durations for telehealth or office visits during the pandemic or postpandemic periods among the clusters of health care facilities.
- H_a_: There are statistically significant differences in patient-to-provider ratios or appointment durations for telehealth or office visits during the pandemic or postpandemic periods among the clusters of health care facilities.

To further explore temporal differences, we compared patient-to-provider ratios and appointment durations within each cluster between the during pandemic and postpandemic periods using paired 2-tailed *t* tests [49]. These tests were also conducted separately by specialty and visit type. The paired 2-tailed *t* test hypotheses are as follows:

- H_0_: There is no statistically significant difference in mean appointment durations or patient-to-provider ratios for telehealth or office visits within each cluster between the during pandemic and postpandemic periods.
- H_a_: There is a statistically significant difference in mean appointment durations or patient-to-provider ratios for telehealth or office visits within each cluster between the during pandemic and postpandemic periods.

Before performing these tests, we assessed the assumptions of normality and homogeneity of variance using the Shapiro-Wilk test and the Levene test [50,51]. If these assumptions were violated, alternative nonparametric methods were used: the Kruskal-Wallis test replaced 1-way ANOVA, and the Wilcoxon signed rank test substituted for the paired 2-tailed *t* test. Post-hoc comparisons were conducted using Tukey honestly significant difference for ANOVA [52] and Dunn test with Bonferroni correction for the Kruskal-Wallis test [53].

## Results

### Overview

This section presents the results in alignment with Figure 2. We begin with an exploratory data analysis to highlight telehealth trends and resource use patterns. We then present the results of the learning algorithm and identify the factors influencing resource use. Next, we describe the clustering of health care facilities and report the comparative analyses of resource use across these clusters.

These results directly address the study objectives by examining how telehealth use and patient- and facility-related factors affect patient-to-provider ratios and appointment durations and by exploring how clusters of health care facilities differ in telehealth adoption and performance metrics over time.

### Trends in Telehealth and Office Visits

#### Overview

Table 2 presents the quarterly number of telehealth and office visits from 2018 to 2023. A significant decline in total visits is observed during the early months of the pandemic (Q1 and Q2 2020), accompanied by a sharp increase in telehealth visits, peaking in Q2 2020. Following this surge, telehealth use gradually declined but remained consistently used, underscoring its sustained role in health care delivery through the end of 2023.

**Table 2:** Number of telehealth and office visits (2018-2023).

Quarter, year	Telehealth visits, n	Office visits, n
Q1, 2018	31,650	38,361,217
Q2, 2018	34,208	39,240,189
Q3, 2018	38,992	38,748,133
Q4, 2018	49,533	40,941,939
Q1, 2019	52,593	43,038,467
Q2, 2019	57,869	44,101,303
Q3, 2019	60,092	44,731,534
Q4, 2019	74,953	46,290,450
Q1, 2020	1,001,360	44,025,023
Q2, 2020	9,566,515	24,844,182
Q3, 2020	6,069,126	39,700,887
Q4, 2020	6,426,342	40,275,185
Q1, 2021	6,562,667	42,052,938
Q2, 2021	4,748,016	47,461,381
Q3, 2021	4,194,784	50,377,723
Q4, 2021	4,142,676	51,070,485
Q1, 2022	5,011,821	51,412,365
Q2, 2022	3,993,359	54,568,671
Q3, 2022	3,874,799	55,658,385
Q4, 2022	4,123,914	57,645,034
Q1, 2023	4,318,727	61,454,472
Q2, 2023	3,909,782	62,379,729
Q3, 2023	3,814,827	61,818,757
Q4, 2023	4,207,164	64,956,975

#### Evaluating Telehealth Use Across Health Care Specialties

**Figure 3:**
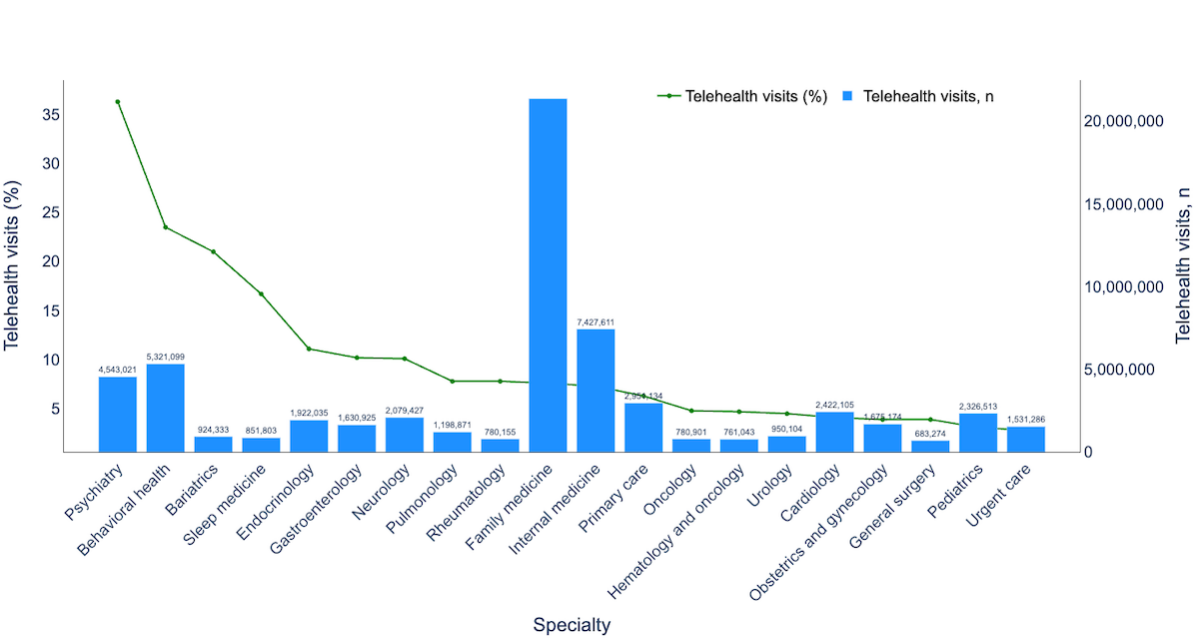
Percentage and number of telehealth visits (2018-2023).

We analyzed health care specialties with the highest telehealth adoption to identify best practices for virtual care (Figure 3). Significant variations were observed, with psychiatry leading telehealth visits at 36.9% (4,543,021/12,311,710), followed by behavioral health (5,321,099/22,264,013, 23.9%), bariatrics (924,333/4,360,061, 21.2%), and sleep medicine (851,803/5,070,256, 16.8%). These adoption rates highlight strong telehealth integration within these specialties, offering valuable insights into factors driving adoption and associated outcomes. Visit trends for these specialties can be seen in Multimedia Appendix 1.

### Data Preprocessing

#### Overview

Table 3 shows the number of visits before and after data preprocessing and outlier removal, broken down by specialty as well as telehealth and office visits. The counts and percentages of visits excluded at each step are provided in Tables S1 and S2 in Multimedia Appendix 2.

**Table 3:** Number of visits before and after data preprocessing and excluding outliers.

Specialty	Telehealth			Office		
	Before data preprocessing, n	After data preprocessing, n	After excluding outliers, n	Before data preprocessing, n	After data preprocessing, n	After excluding outliers, n
						
Psychiatry	4,264,891	2,928,491	2,405,428	7,331,621	4,922,991	4,053,490
Behavioral health	5,241,929	3,977,131	3,361,661	16,527,421	11,699,435	9,589,019
Bariatrics	897,721	664,836	497,262	3,372,671	2,650,826	2,234,762
Sleep medicine	839,368	588,947	405,204	4,168,268	3,152,911	2,737,440

#### Proportion of Telehealth Visits Over Time Across Rural and Urban Areas

**Figure 4:**
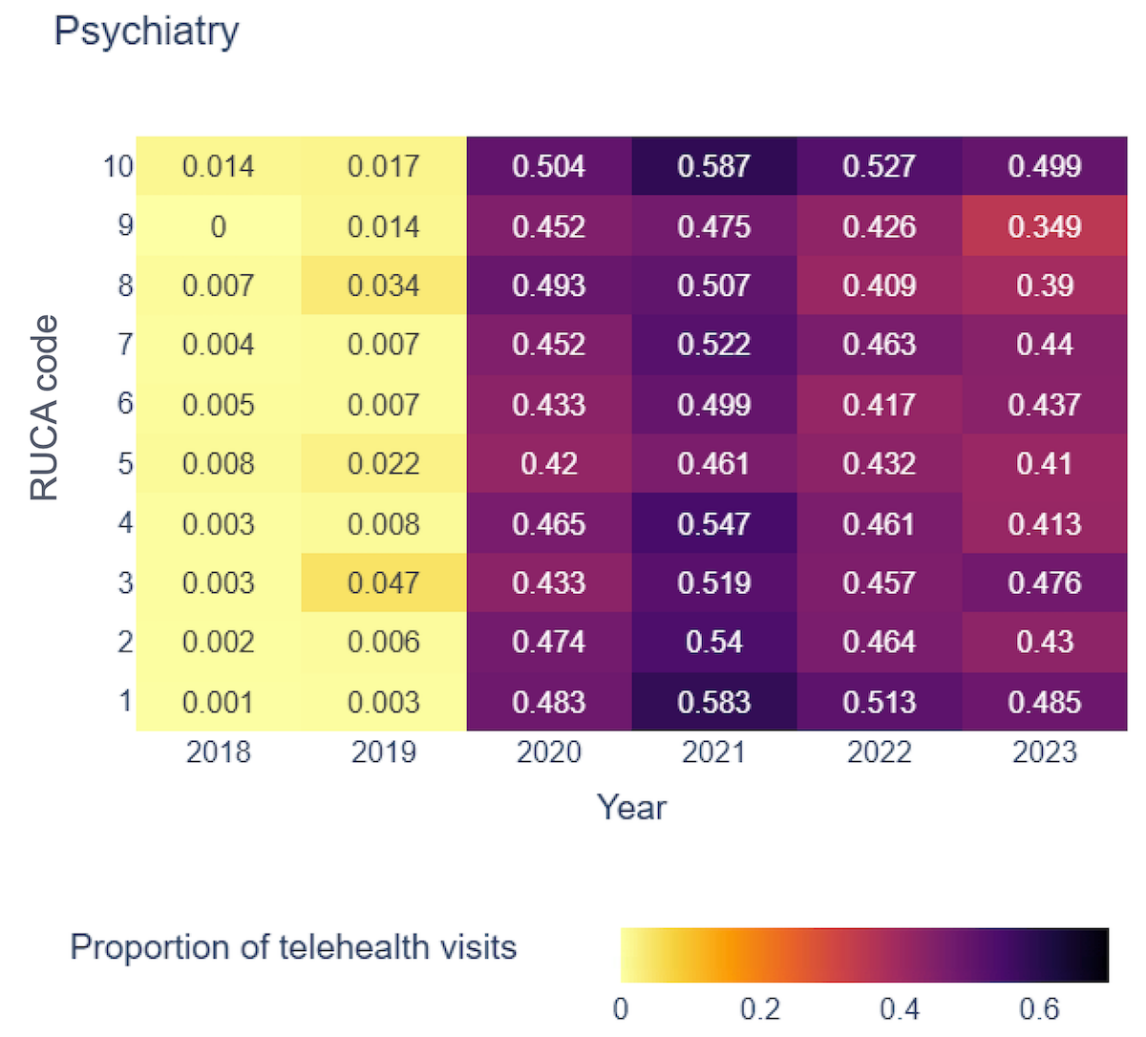
Proportion of telehealth visits over time in psychiatry. RUCA: rural-urban commuting area.

**Figure 5:**
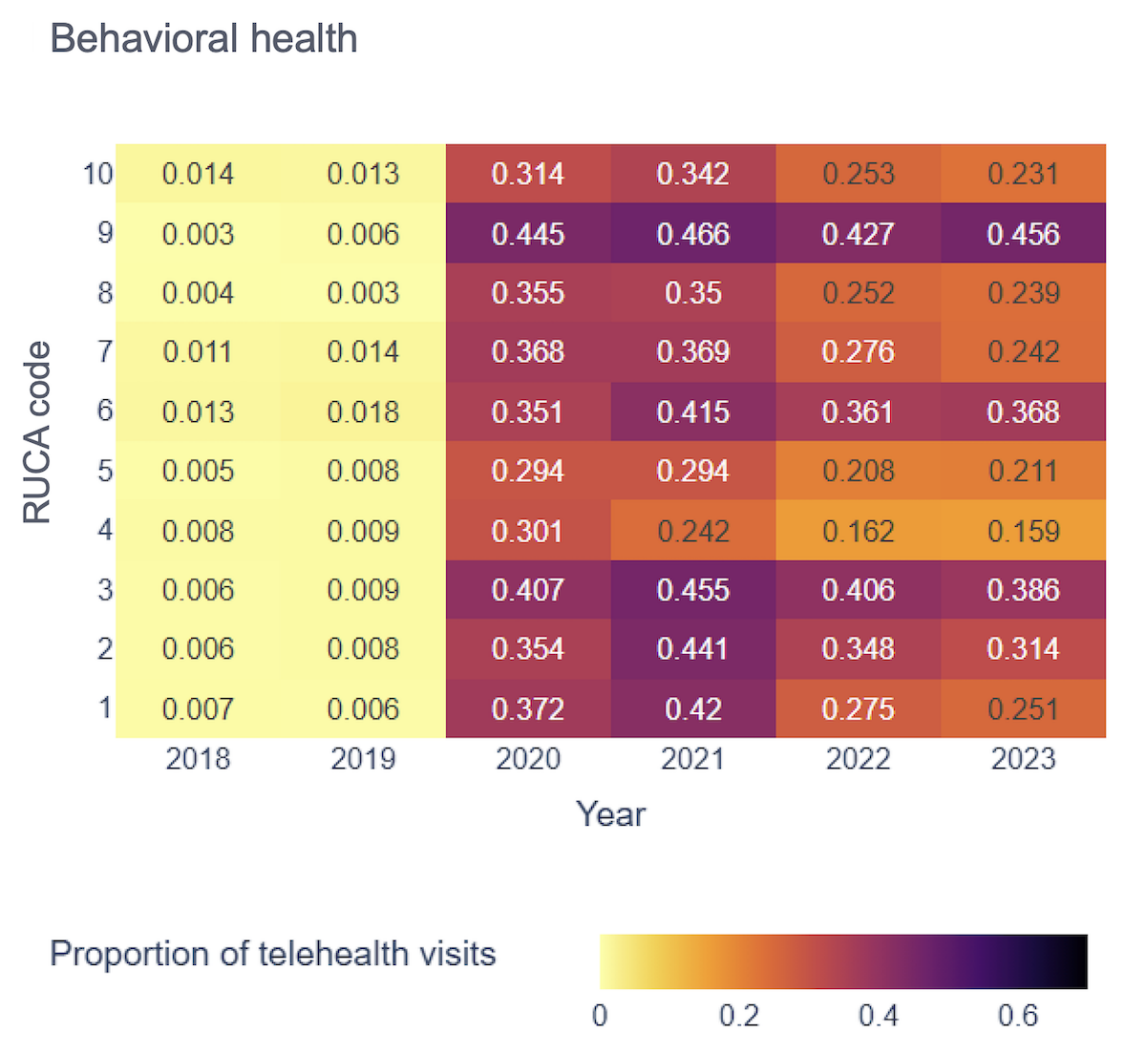
Proportion of telehealth visits over time in behavioral health. RUCA: rural-urban commuting area.

**Figure 6:**
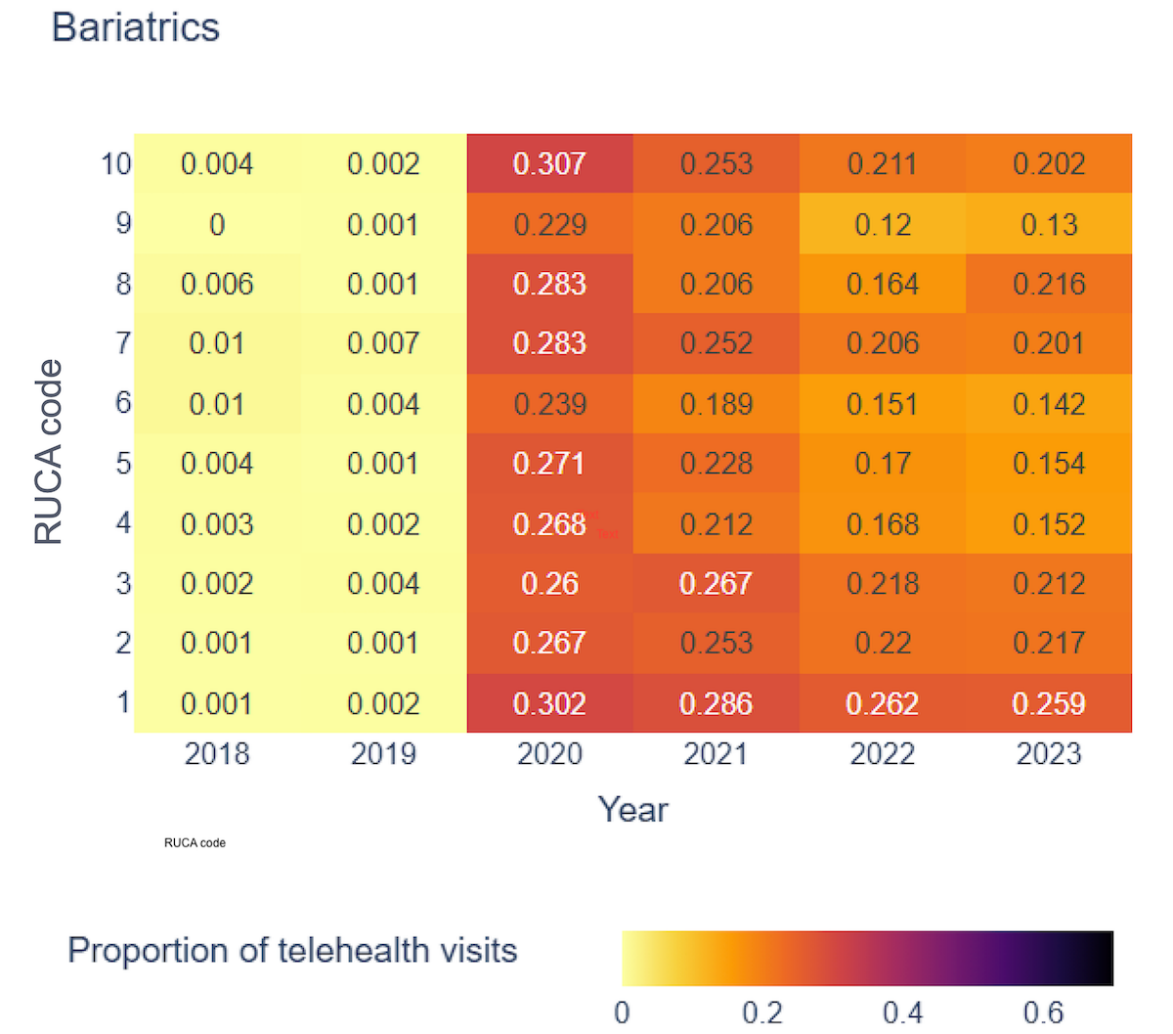
Proportion of telehealth visits over time in bariatrics. RUCA: rural-urban commuting area.

**Figure 7:**
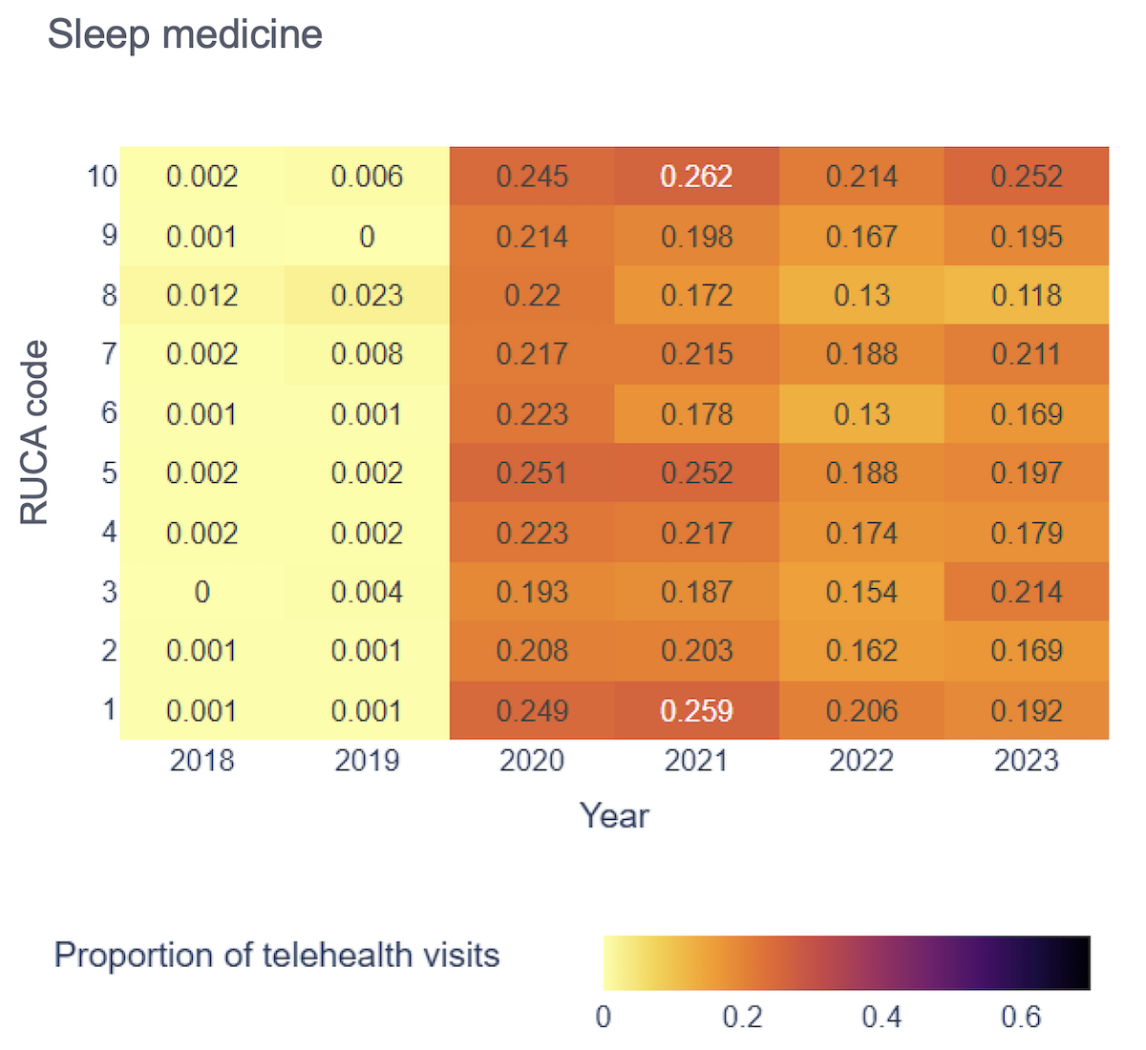
Proportion of telehealth visits over time in sleep medicine. RUCA: rural-urban commuting area.

Figures 4-7 display the proportion of telehealth visits across RUCA codes from 2018 to 2023 for the selected 4 specialties.

In psychiatry, telehealth use was minimal before 2020 but increased sharply, particularly in rural areas (RUCA codes 8 and 10). The highest use occurred in 2021, with RUCA code 10 reaching 0.587 and RUCA code 1 at 0.583. RUCA code 10 maintained the highest telehealth use from 2021 to 2023, with RUCA code 1 consistently following. This trend highlights substantial telehealth adoption in psychiatry across both highly rural and urban areas. Behavioral health exhibited a similar trajectory, with negligible telehealth use before 2020. RUCA code 9 peaked at 0.466 in 2021, underscoring telehealth’s significance in areas with low commuting rates [54].

In bariatrics, telehealth adoption began in 2020, with RUCA code 10 showing the highest rate (0.307). From 2021 to 2023, RUCA code 1 led telehealth use, reflecting its essential role in both urban and rural settings for bariatric services. Sleep medicine experienced gradual growth in telehealth adoption, with RUCA code 10 reaching its highest rate (0.262) in 2021. By 2023, telehealth use in rural areas remained high.

Our analysis revealed a consistent upward trend in telehealth visits across all 4 specialties from 2018 to 2023, particularly pronounced in specific RUCA codes. This underscored the growing acceptance and importance of telehealth services in both the most urban and rural areas. Although telehealth use peaked during the pandemic, it remained widely adopted across regions. However, the absence of uniform patterns across all RUCA codes for every specialty highlighted the unique telehealth use trends within each specialty, emphasizing the need for tailored analyses.

#### Resource Use Over Time

**Figure 8:**
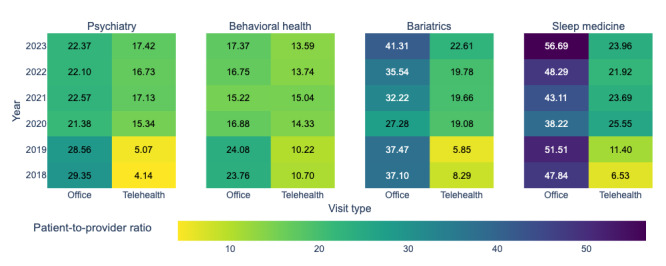
Patient-to-provider ratio over time.

Figure 8 illustrates the trends in patient-to-provider ratios for telehealth and office visits across specialties from 2018 to 2023. The COVID-19 pandemic in 2020 caused a marked increase in telehealth ratios, as the health care system adapted to unprecedented challenges. In the postpandemic period, these ratios either stabilized or continued to grow, reflecting sustained telehealth adoption and ongoing adjustments in provider practices.

**Figure 9:**
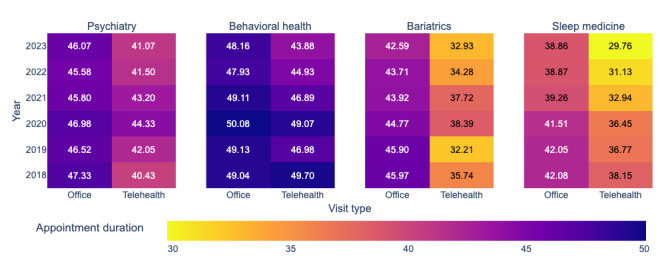
Appointment durations over time.

Figure 9 highlights appointment duration trends over the same period. During the pandemic, telehealth appointments were generally longer but eventually stabilized or decreased in the postpandemic period. Telehealth appointments were consistently shorter than office visits, with the exception of behavioral health in 2018.

Distinct trends across specialties underscore the necessity of analyzing factors influencing patient-to-provider ratios and appointment durations. The substantial variations during and after the pandemic also call for a detailed comparison of these periods.

Given the large number of health care facilities in this study, we applied a clustering approach to group facilities with similar characteristics, allowing more comprehensive comparisons and clearer interpretation of patterns. Building on this, we developed models to examine resource use, its influencing factors, followed by a comparative analysis.

### Learning Algorithms

To analyze the impact of patient- and facility-related factors on resource use, we evaluated 3 ML regression models, including random forest, XGBoost, and DNN models (see Tables S1-S3 in Multimedia Appendix 3 for model performances and hyperparameters). Ultimately, XGBoost was chosen due to its superior predictive performance.

#### XGBoost Regression Model

Separate models were developed for each specialty, with hyperparameters optimized using Optuna. Typically, data were divided into an 80% training and 20% testing split to balance learning and evaluation [55]. However, to improve model performance, we adjusted the splits to 90%-10% split based on *R*^2^ results. Table 4 shows the number of training and testing rows per specialty.

**Table 4:** Training and testing splits for the models.

Specialty	Training	Testing
Psychiatry	9335	1038
Behavioral health	8464	941
Bariatrics	8892	989
Sleep medicine	9427	1049

*R*^2^ values were calculated to evaluate model performance, showing strong predictive power for patient-to-provider ratios and moderate power for appointment durations (Table 5). All results were obtained within 4 seconds.

**Table 5:** R2 values for extreme gradient boosting regression models.

Specialty	Patient-to-provider ratio, *R*^2^	Appointment duration, *R*^2^
Psychiatry	0.9935	0.6923
Behavioral health	0.9689	0.6673
Bariatrics	0.9948	0.6519
Sleep medicine	0.9934	0.6101

#### Feature Importance

In SHAP summary plots, red points indicate higher feature values and blue points indicate lower values; their position on the x-axis shows whether they increase (right) or decrease (left) the prediction.

**Figure 10:**
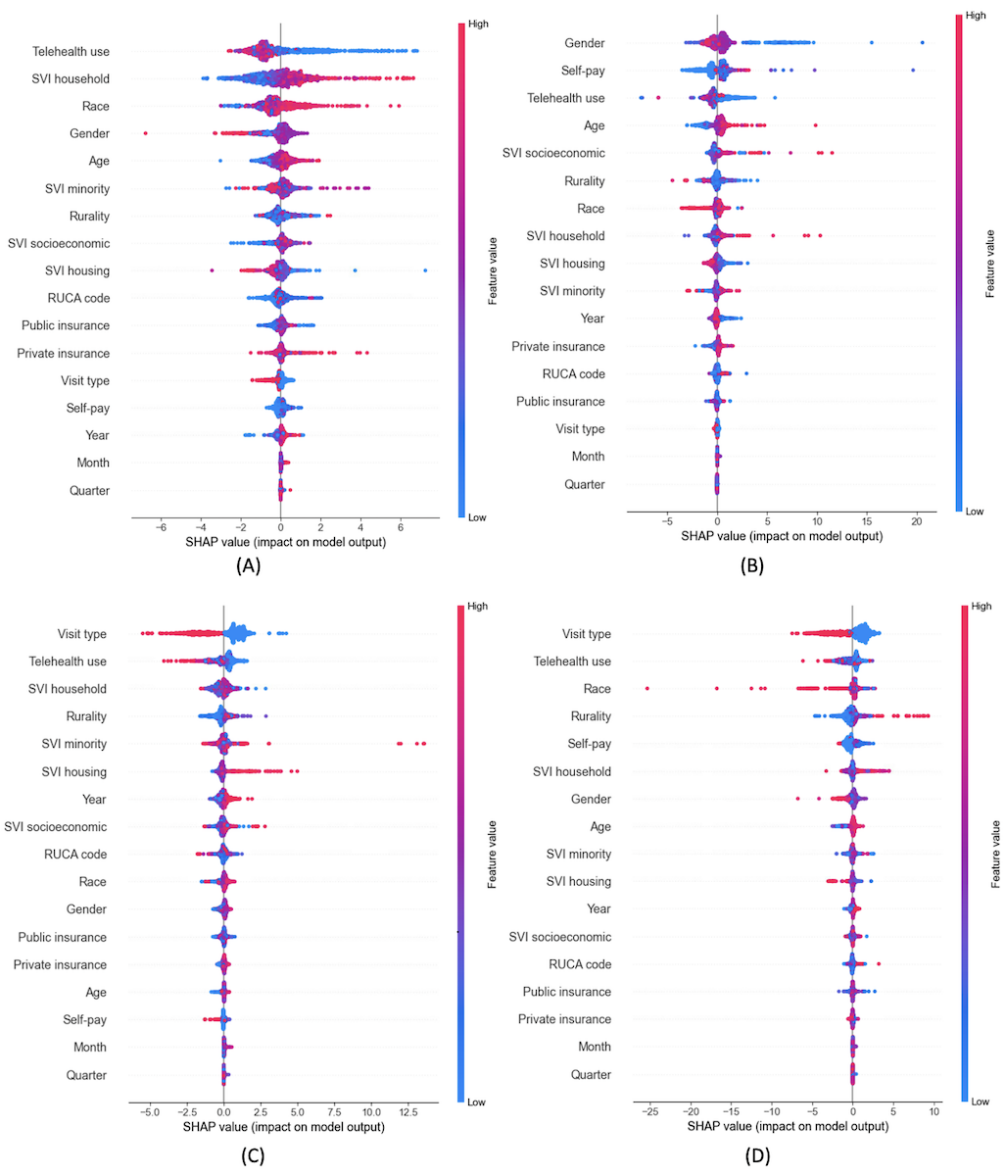
SHAP summary plots—patient-to-provider ratio. (A) Psychiatry, (B) behavioral health, (C) bariatrics, and (D) sleep medicine. RUCA: rural-urban commuting area; SHAP: Shapley Additive Explanations; SVI: Social Vulnerability Index.

Figures 10A-10D displays SHAP summary plots for output 1 (patient-to-provider ratio) across the selected specialties. Across all specialties, telehealth use emerges as an important factor, with higher telehealth use generally resulting in fewer patients per provider.

In psychiatry, SVI household and race significantly influence patient loads, reflecting socioeconomic and demographic factors. Higher SVI household values correspond to increased patient loads in psychiatry, behavioral health, and sleep medicine, while in bariatrics, no consistent trend is observed. Similarly, race shows mixed effects: health care facilities with a higher proportion of visits by White patients (red points) are linked to more patients per provider in psychiatry, but with fewer patients per provider in behavioral health and sleep medicine.

In behavioral health, sex is a significant factor, with facilities having a lower proportion of visits by female patients (blue points) associated with higher patient loads. Self-pay is also significant; however, it shows mixed effects. In bariatrics, visit type emerges as the most critical feature, with telehealth visits (red points) generally associated with fewer patients per provider compared to office visits (blue points), similar to sleep medicine. Telehealth use is the second most influential factor, where higher telehealth adoption correlates with decreased patient loads, again consistent with findings in sleep medicine.

In summary, while features such as telehealth use, visit type, SVI household, and race are universally important, the varying influence of demographic and socioeconomic factors underscores the diverse drivers of patient distribution across specialties.

**Figure 11:**
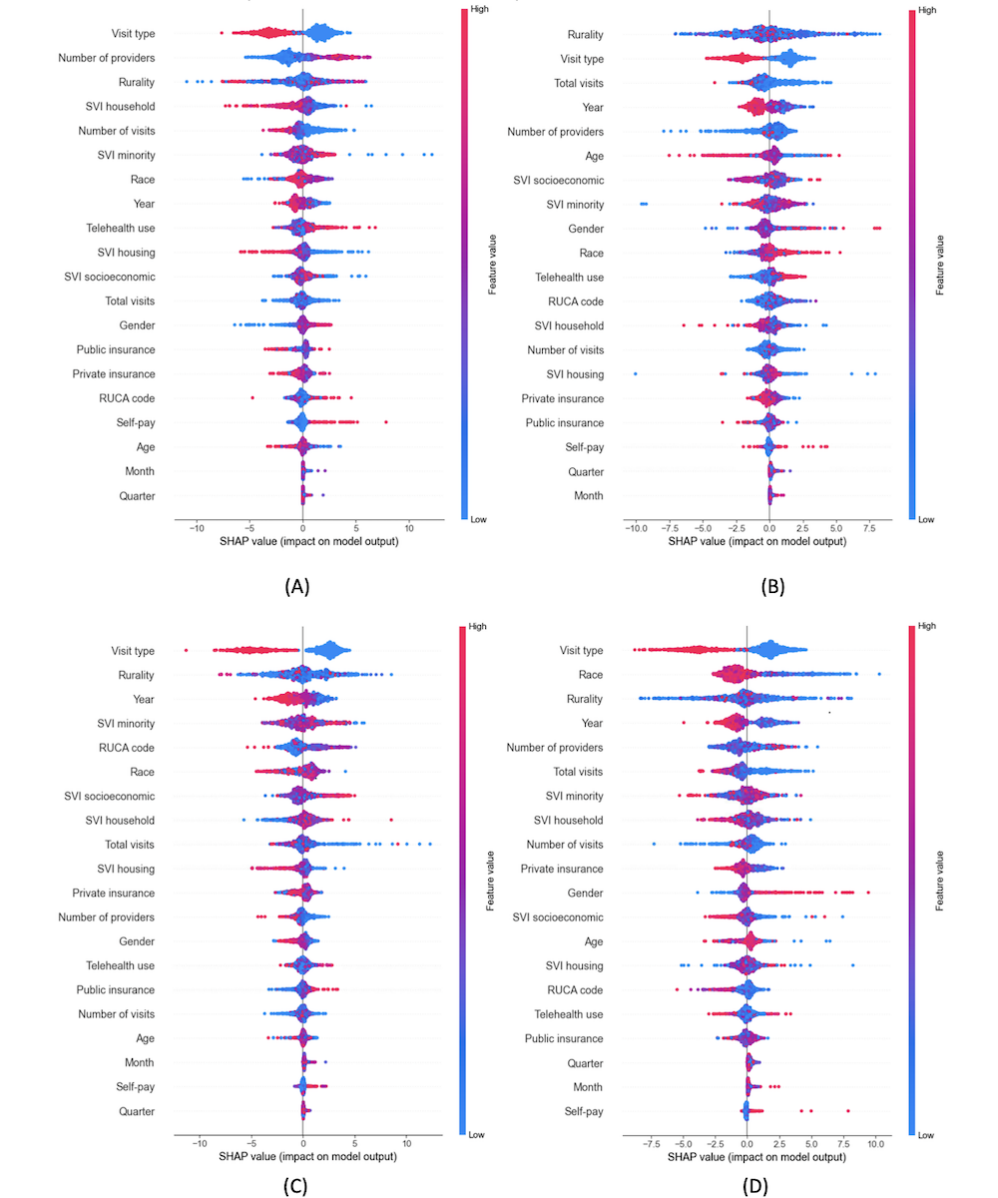
SHAP summary plots—appointment duration. (A) Psychiatry, (B) behavioral health, (C) bariatrics, and (D) sleep medicine. RUCA: rural-urban commuting area; SHAP: Shapley Additive Explanations; SVI: Social Vulnerability Index.

Figure 11A-11D displays SHAP summary plots for output 2 (appointment durations). Across specialties, visit type is an important factor: telehealth visits typically result in shorter durations, while office visits are associated with longer durations. Rurality also emerges as a significant factor but exhibits mixed effects across the 4 specialties. Year is another influential variable, with higher values (2021-2023) linked to shorter appointment durations, and lower values (2018-2020) linked to longer durations.

In psychiatry, facilities with more providers (larger health care facilities) tended to have longer appointment durations, whereas the opposite pattern was observed in bariatrics, and no clear trend was evident in behavioral health or sleep medicine. Race is particularly important in sleep medicine, where facilities with a higher proportion of visits by White patients (red points) are associated with shorter durations.

Overall, visit type, rurality, and year consistently emerge as the most influential factors for appointment durations. Telehealth visits generally lead to shorter appointments, more recent years are associated with reduced durations, and rurality shows mixed effects across specialties.

### Clustering Model

#### Overview

Our clustering model selected key features using SHAP analysis across all 4 health care specialties. To ensure diversity, we included 1 feature from each major category: 1 SVI metric, 1 demographic variable, 1 representing facility size, and 1 related to insurance. The final set of features consisted of number of providers, telehealth use, RUCA code, SVI socioeconomic, and public insurance. We applied the isolation forest algorithm with a contamination rate of 0.15 (excluding 15% of data points as anomalies). Table 6 summarizes the number of health care facilities in each specialty before and after data cleaning.

**Table 6:** Number of health care facilities within the clustering model.

Specialty	Before cleaning, n (%)	After cleaning, n (%)	Clusters created
Psychiatry	134 (24.9)	114 (24.9)	6
Behavioral health	143 (26.5)	121 (26.5)	6
Bariatrics	117 (21.7)	99 (21.7)	6
Sleep medicine	145 (26.9)	123 (26.9)	6

After evaluating clustering metrics, we selected 6 clusters for each specialty. Please see Figure S1A-S1D and Table S1 in Multimedia Appendix 4 and Multimedia Appendix 5 for details on the evaluation of clustering metrics and the importance of factors in the clustering model. Each cluster represents a distinct group of health care facilities. Figure S1A-S1D in Multimedia Appendix 6 displays the average telehealth use, rurality, SVI socioeconomic, SVI minority, and number of providers across clusters for each specialty. Figure S2A-S2D in Multimedia Appendix 6 illustrates the average patient-to-provider ratio, appointment durations, and facility counts per cluster. Additional feature averages for each cluster are detailed in Tables S1-S4 in Multimedia Appendix 6.

#### Clusters of Health Care Facilities

Each specialty’s clusters display unique resource use and patient care characteristics, emphasizing the diverse nature of health care delivery. This analysis highlights opportunities for optimizing health care services for various patient populations and settings.

In Textbox 2, we summarize the clustering results across specialties, focusing on average feature values, resource use, and variations observed during and after the pandemic.

**Highlights by specialty and clusters.**

**Psychiatry**

Telehealth appointments were shorter than office visits, with cluster 6 having the highest telehealth use.

- Cluster 1: Younger patients (average age 40.68 years).
- Cluster 2: Few providers, 27.0% (5647/20,915) telehealth use, high rurality, and low telehealth patient-to-provider ratio during the pandemic, which increased after the pandemic with short telehealth appointments.
- Cluster 3: High public insurance coverage due to older patients (average age 49.8 years).
- Cluster 4: Many providers, 40.0% (48,585/121,463) telehealth use, low rurality, long appointments, and low patient-to-provider ratio.
- Cluster 5: High patient-to-provider ratio, 25.0% (7691/30,764) telehealth use, and vulnerable population based on the following factors: SVI household, housing, minority, and socioeconomic.
- Cluster 6: Few providers, 88.0% (10,607/12,053) telehealth use, low rurality, high self-paid visits, short appointments, and the lowest patient-to-provider ratio.

**Behavioral health**

After the pandemic, office visits saw higher patient-to-provider ratios and shorter durations, while telehealth ratios were lower.

- Cluster 1: High public insurance coverage.
- Cluster 2: Lowest telehealth use, 9.0% (1813/20,144), low telehealth, and high office patient-to-provider ratios.
- Cluster 3: Few providers, 83.0% (12,870/15,506) telehealth use, long appointments, and the highest patient-to-provider ratio.
- Cluster 4: Most providers and visits, low telehealth patient-to-provider ratio, and moderate telehealth use, 23.0% (39,890/173,435).
- Cluster 5: Moderate providers, 20.0% (18,533/92,665) telehealth use, high rurality, the highest office patient-to-provider ratio, and short telehealth appointments.
- Cluster 6: Few providers, 55.0% (3544/6444) telehealth use, and high self-paid visits.

**Bariatrics**

Telehealth appointments were shorter than office visits, with 80.0% (119,241/149,051) of visits from female patients.

- Cluster 1: Few providers, 7.0% (2047/29,243) telehealth use, and high rurality.
- Cluster 2: Moderate providers, 42.0% (9888/23,543) telehealth use, and the highest postpandemic telehealth patient-to-provider ratio.
- Cluster 3: Older patients (average age 50.91 years), least vulnerable, 15.0% (3895/25,967) telehealth use, and short appointments.
- Cluster 4: Few providers, 13.0% (1057/8131) telehealth use, the youngest patients, and high public insurance coverage.
- Cluster 5: Most providers, stable telehealth ratios, and increased office patient-to-provider ratio after the pandemic.
- Cluster 6: Few providers, 5.0% (793/15,860) telehealth use, lowest telehealth, and highest office patient-to-provider ratio.

**Sleep medicine**

After the pandemic, office visits had higher patient-to-provider ratios, telehealth ratios were lower, and sleep medicine had an older patient population with 50.0% (98,312/196,624) female patients.

- Cluster 1: Younger, vulnerable patients, low public insurance, 19.0% (2024/10,651), low telehealth use, 5.0% (1482/29,640), and long appointments.
- Cluster 2: Vulnerable patients and low telehealth use, 7.0% (1747/24,957).
- Cluster 3: High public insurance coverage.
- Cluster 4: Moderate providers, 15.0% (3737/24,913) telehealth use, high rurality, short appointments, and high patient-to-provider ratio.
- Cluster 5: Most providers and visits, low telehealth 9.0% (9171/101,900), and a high female patient proportion.
- Cluster 6: Moderate providers, 47.0% (13,282/28,260) telehealth use, short appointments, high telehealth, and low office patient-to-provider ratios.

### Comparative Analysis

#### Overview

This section examines differences in patient-to-provider ratios and appointment durations between telehealth and office visits during and after the pandemic across specialty-specific clusters. Tables S1 and S1 in Multimedia Appendix 7 provide detailed data on these metrics, with Figures S1A-S1D and S2A-S2D in Multimedia Appendix 7 visualizing these comparisons. A significance threshold of α=.05 was used to assess differences in resource use over time.

#### Differences Between Clusters

All *P* values obtained are less than .05, indicating that the observed differences in patient-to-provider ratios (or appointment durations) are statistically significant. In addition, we present Dunn test results with corresponding *P* values in Tables S1-S8 in Multimedia Appendix 8. For instance, Table S1 in Multimedia Appendix 8 shows significant differences in telehealth patient-to-provider ratios during the pandemic between cluster 6 and clusters 1-5. After the pandemic, additional differences emerged, such as between cluster 1 and clusters 2 and 3, indicating varying resource allocation and patient needs.

#### Resource Use During and After the Pandemic

Table 7 summarizes these results. In psychiatry, telehealth appointment durations significantly changed (*P*=.03), while other metrics remained stable. Behavioral health showed significant differences in office visit metrics (*P*=.01) and appointment durations for both telehealth (*P*=.02) and office visits (*P*=.03). Bariatrics exhibited significant changes in telehealth visit patient-to-provider ratios (*P*<.001), and office appointment durations (*P*=.02). Sleep medicine showed marginal significance in telehealth patient-to-provider ratios (*P*=.08) and significant changes in office visit patient-to-provider ratios (*P*<.001). These results underscore the pandemic’s varied impact on resource use across specialties and visit types, particularly in behavioral health.

**Table 7:** Hypothesis testing results for patient-to-provider ratios and appointment durations during and after the pandemic for telehealth and office visit^a^.

Specialty	During vs post			
	Patient-to-provider ratio		Appointment durations	
	H_a_: μ^d,t,ptp^≠μ^p,t,ptp^, *P* value	H_a_: μ^d,o,ptp^≠μ^p,o,ptp^, *P* value	H_a_: μ^d,t,app^≠μ^p,t,app^, *P* value	H_a_: μ^d,o,app^≠μ^p,o,app^, *P* value
Psychiatry	.96	.57	.03	.91
Behavioral health	.25	.01	.02	.03
Bariatrics	.92	*<*.001	.02	.86
Sleep medicine	.08	*<*.001	.10	.95

^a^μ^d,t,ptp^, μ^p,t,ptp^: mean patient-to-provider ratio for telehealth visits during the pandemic and after the pandemic; μ^d,o,ptp^, μ^p,o,ptp^ mean patient-to-provider ratio for office visits during the pandemic and after the pandemic; μ^d,t,app^, μ^p,t,app^: mean appointment duration for telehealth visits during the pandemic and after the pandemic; and μ^d,o,app^, μ^p,o,app^: mean appointment duration for office visits during the pandemic and after the pandemic.

## Discussion

### Principal Findings

This study analyzed telehealth adoption using a nationwide dataset, focusing on psychiatry, behavioral health, bariatrics, and sleep medicine—specialties with high telehealth use. Our analysis shows that telehealth use peaked in 2020 with the COVID-19 pandemic and, while it has declined since, it remains above prepandemic levels. Telehealth adoption was especially high in both the most urban and most rural areas, underscoring telehealth’s role in addressing both access and convenience. Importantly, telehealth continues to be in use, highlighting its lasting role in health care delivery.

In terms of resource use, telehealth visits were typically associated with shorter appointment durations, while patient-to-provider ratios varied across specialties and over time. These findings support our first hypothesis and highlight telehealth’s role in improving time efficiency, though its effects on patient load vary across specialties. By combining descriptive, predictive, and clustering analyses, our study offers an integrated understanding of how facility characteristics, patient demographics and socioeconomic factors, and telehealth adoption jointly shape resource use patterns.

Identifying drivers of resource is essential for informing policy and management decisions aimed at optimizing resources, staffing, reimbursement, and equitable access to care. To examine variation in resource use among health care facilities, we clustered similar health care facilities and then compared these clusters. This approach allowed us to generate insights at the group level rather than for individual facilities, where substantial variability might otherwise obscure broader patterns.

Our XGBoost regression model provided a robust analytical framework for examining resource use, capturing both facility- and patient-level factors as well as specialty-specific dynamics. Consistent with our second hypothesis, the model results demonstrate that both patient and facility characteristics significantly impact how health care resources are distributed and used.

Socioeconomic and demographic characteristics distinctly influenced resource use, pointing to the need for tailored strategies across facilities, supporting our second hypothesis. For example, higher SVI household values were associated with increased patient loads in psychiatry, behavioral health, and sleep medicine, while no consistent trend was observed in bariatrics. This suggests that facilities serving populations with greater household vulnerability, such as those with higher proportions of older adults, children, single-parent households, or persons with disabilities, may face greater demand pressures. To respond effectively, such facilities could consider targeted staffing adjustments, optimized resource allocation strategies, and supportive services to reduce provider strain and maintain equitable access to care. Additional steps could include improving coordination among providers, expanding telehealth to reduce in-person delays, training providers to better serve vulnerable populations, and advocating for policies or reimbursement models that reflect the higher resource needs of these facilities.

Race played a complex role in resource use, with effects varying across specialties. Facilities serving a higher proportion of White patients experienced heavier patient loads in psychiatry but lighter loads in behavioral health and sleep medicine. These contrasting patterns may reflect differences in access, care-seeking behaviors, or service delivery models across patient populations. Recognizing these disparities is important for designing culturally responsive interventions, expanding outreach in underserved groups, and ensuring that telehealth integration supports equitable care delivery.

Beyond sociodemographic influences, the level of telehealth adoption was itself an important determinant of resource use. Facilities with higher telehealth use generally had fewer patients per provider. This finding further supports our first hypothesis and suggests that telehealth can reduce patient load, helping health care facilities manage workloads more effectively and achieve a more balanced distribution of care. To capitalize on this potential, facilities could expand telehealth offerings to mitigate provider restrictions during peak demand and implement scheduling systems that direct appropriate cases to telehealth. In addition, investing in provider training and digital infrastructure can ensure that reduced patient loads translate into improved care quality rather than inefficiencies. Policy support for telehealth reimbursement can further encourage its integration into routine practice.

Telehealth visits were typically shorter in duration than office visits, largely due to the minimization of administrative tasks and the absence of physical examinations. Shorter appointments do not necessarily indicate lower quality of care and should not be viewed as a trigger for additional follow-ups. In fact, a study by Epic Research found that follow-up visits were less common after telehealth visits than after office visits across specialties such as psychiatry, behavioral health, bariatrics, and sleep medicine, suggesting that telehealth can be effective even with shorter durations [56]. Moreover, telehealth visits are often billed at lower levels of service than office visits within the same specialty, reflecting the simpler nature of these visits [57].

The number of providers in a facility also influenced appointment durations, but the effects differed by specialty. In psychiatry, larger facilities with more providers were linked to longer appointments, possibly due to the added complexity of coordinating care or the flexibility to spend more time with each patient. In contrast, bariatric facilities with more providers tended to have shorter appointments, which may result from greater specialization or streamlined workflows. These patterns show that facility size and staffing models affect how care is delivered and how time is allocated, shaping the role telehealth can play across different specialties. These results support our second hypothesis, indicating that organizational capacity and structure play an important role in resource use.

Additionally, supporting our third hypothesis, our clustering and comparative analyses showed clear differences among health care facilities in terms of patient demographics, resources, and telehealth adoption. These patterns highlight that health care delivery varies widely depending on facility characteristics and patient populations, meaning a single strategy will not work for all facilities. Therefore, policies should be tailored to the specific needs of different types of facilities. Facilities serving vulnerable populations may require additional funding, staffing, and stronger reimbursement policies to avoid overloading providers and to ensure fair access to care. Small facilities with few providers but high telehealth use would benefit from investments in digital infrastructure and training. Larger facilities with more providers but longer appointment times (such as in psychiatry) may need stronger care coordination systems and efficiency measures to ensure that time is used effectively. Rural facilities, where shorter appointments may reflect efficiency pressures, could benefit from standardized protocols and resource-sharing networks to balance time with quality. Specialties with low telehealth adoption, such as some bariatrics and sleep medicine clusters, should receive targeted support to address barriers like broadband access, patient acceptance, or reimbursement issues.

Sharing best practices from clusters with high telehealth adoption can guide regions with lower adoption, ensuring equitable access and improved service quality. Our findings emphasize the need for specialty-specific telehealth integration, supportive reimbursement models, and user-friendly platforms. Investing in telehealth literacy, infrastructure, and tailored policies will optimize resource use and enhance patient outcomes across diverse settings.

### Limitations

A key limitation of this study is the inability to directly analyze broadband access, a well-established determinant of telehealth use [13], because the Cosmos dataset does not keep track of broadband availability. However, prior research indicates that SVI themes are strongly associated with broadband access and adoption [58]. As such, while we cannot analyze broadband directly, the use of SVI themes provides a meaningful proxy for understanding disparities in telehealth access. Figure S1 in Multimedia Appendix 9 [59] provides an overview of county-level RUCA codes, and Figure S2 in Multimedia Appendix 9 shows declining broadband subscription rates with increasing RUCA codes, further supporting the relevance of our rurality and vulnerability measures in capturing connectivity-related barriers.

Another limitation relates to the potential selection bias stemming from the use of Epic’s Cosmos dataset. Although Epic is used by a diverse range of health care organizations, it tends to be more commonly adopted by larger health systems. This could limit the generalizability of our findings to smaller or independent providers. We believe that clustering health care facilities based on multiple factors—including the number of providers—helped mitigate this issue by grouping facilities with similar characteristics, thereby enabling more balanced comparisons.

Additionally, the randomized date shifting in the Cosmos dataset for confidentiality, with patient visit dates adjusted by 0 to 30 days, may affect temporal precision. However, the small shift does not significantly impact the overall findings. Additionally, the absence of zip code information for health care facilities limits certain geographic analyses. Using a smaller subset of facilities could address this but would reduce population coverage. Nonetheless, Cosmos mitigates the complexity of diverse electronic health record systems by using a standardized system, ensuring consistent data processing across facilities.

### Conclusions

This study presents a comprehensive analysis of telehealth adoption and resource use across psychiatry, behavioral health, bariatrics, and sleep medicine using a nationwide dataset. Our findings highlight significant shifts in telehealth and office visits during and after the COVID-19 pandemic, with notable regional and specialty-specific variations. By examining the impact of telehealth use, patient- and facility-level factors on resource use, and identifying facility clusters, this study provides a multifaceted understanding of telehealth’s role in shaping health care delivery. Consistent with our first hypothesis, telehealth visits were associated with shorter appointment durations. However, patient-to-provider ratios varied across visit types, reflecting the diverse ways telehealth influences resource use.

The second and third hypotheses were also supported. Patient- and facility-level factors significantly impacted resource use, while clustering revealed distinct groups of health care facilities with varying levels of telehealth adoption and resource use. These findings underscore that resource use is shaped not only by technology adoption but also by demographic, socioeconomic, and organizational factors. For example, sociodemographic characteristics, such as household vulnerability and race, impacted patient loads and appointment durations, suggesting the need for equity-focused policies. Facility size also played a role, indicating that organizational capacity must be considered when planning telehealth integration. Clustering further revealed distinct facility profiles, showing that telehealth strategies must be tailored to local contexts rather than relying on universal approaches.

Telehealth not only enhances health care efficiency but also offers opportunities to reshape care models and policies. For example, maintaining high telehealth capacity in psychiatry and behavioral health could help address workforce shortages, while in bariatrics, virtual follow-ups can support long-term weight management. In sleep medicine, telehealth can be used for screening and treatment monitoring, complemented by office visits for diagnostic testing. Integrating telehealth into primary and specialty care workflows can expand access, especially for underserved populations, while necessitating policy updates to support equitable infrastructure investments and tailored reimbursement models.

As a valuable tool for improving accessibility and efficiency, telehealth enables health care systems to balance quality, cost, and access. Overall, these findings confirm that telehealth can improve efficiency when guided by specialty-specific strategies. Implementing customized strategies, such as targeted training for providers in specialties with lower telehealth readiness, region-specific infrastructure support, and specialty-appropriate virtual care protocols, can better align telehealth services with patient and provider needs. Addressing specialty- and region-specific needs can optimize telehealth services, improve patient outcomes, and ensure sustainable growth. The use of a nationwide dataset strengthens these findings and provides a solid foundation for future research. Future studies should explore the evolving dynamics of telehealth use and evaluate the effectiveness of these targeted interventions, translating insights into actionable strategies for policymakers, providers, and health care administrators.

## Data Availability

All data generated during this study are included in this published paper and its supplementary information files. However, the data used in the analysis came from Epic Cosmos and are not available for public use. Access to the Epic Cosmos dataset requires affiliation with a participating health organization, as it is a restricted Health Insurance Portability and Accountability Act (HIPAA)–defined limited dataset.

## Appendix

Multimedia Appendix 1 Number of telehealth visits for some specialties.

Multimedia Appendix 2 Data preprocessing steps.

Multimedia Appendix 3 Model performance on resource use metrics by specialty.

Multimedia Appendix 4 Clustering evaluation metrics.

Multimedia Appendix 5 Importance of features in the clustering model.

Multimedia Appendix 6 Summary results of clustering.

Multimedia Appendix 7 Resource use during and after the pandemic.

Multimedia Appendix 8 Statistical differences between clusters.

Multimedia Appendix 9 Broadband subscription rates by rural-urban commuting area codes.

## Abbreviations

DNN: deep neural network
ML: machine learning
RUCA: rural-urban commuting area
SHAP: Shapley Additive Explanations
SVI: Social Vulnerability Index
XGBoost: extreme gradient boosting
