# Supplementary material for: Resource Use Patterns in US Telehealth Services: Machine Learning and Clustering Analysis Across 4 Specialties
Source: JMIR Med Inform. 2026 May 7;14:e78030. doi: 10.2196/78030 (PMC13195373; doi:10.2196/78030)
Supplement: Multimedia Appendix 1 [file medinform_v14i1e78030_app1.docx]

Figure S1 illustrates telehealth visit trends across psychiatry, behavioral health, bariatrics, sleep medicine, family Medicine, and internal medicine. Before the pandemic (2018–2019), telehealth use was minimally in these specialties. During the pandemic (2020–2021), visits surged, with psychiatry and behavioral health surpassing a million visits annually. Family medicine and internal medicine exceeded five million visits annually, while bariatrics and sleep medicine also saw substantial increases. Post-pandemic (2022–2023), telehealth visits remained high, underscoring its lasting role in healthcare delivery.


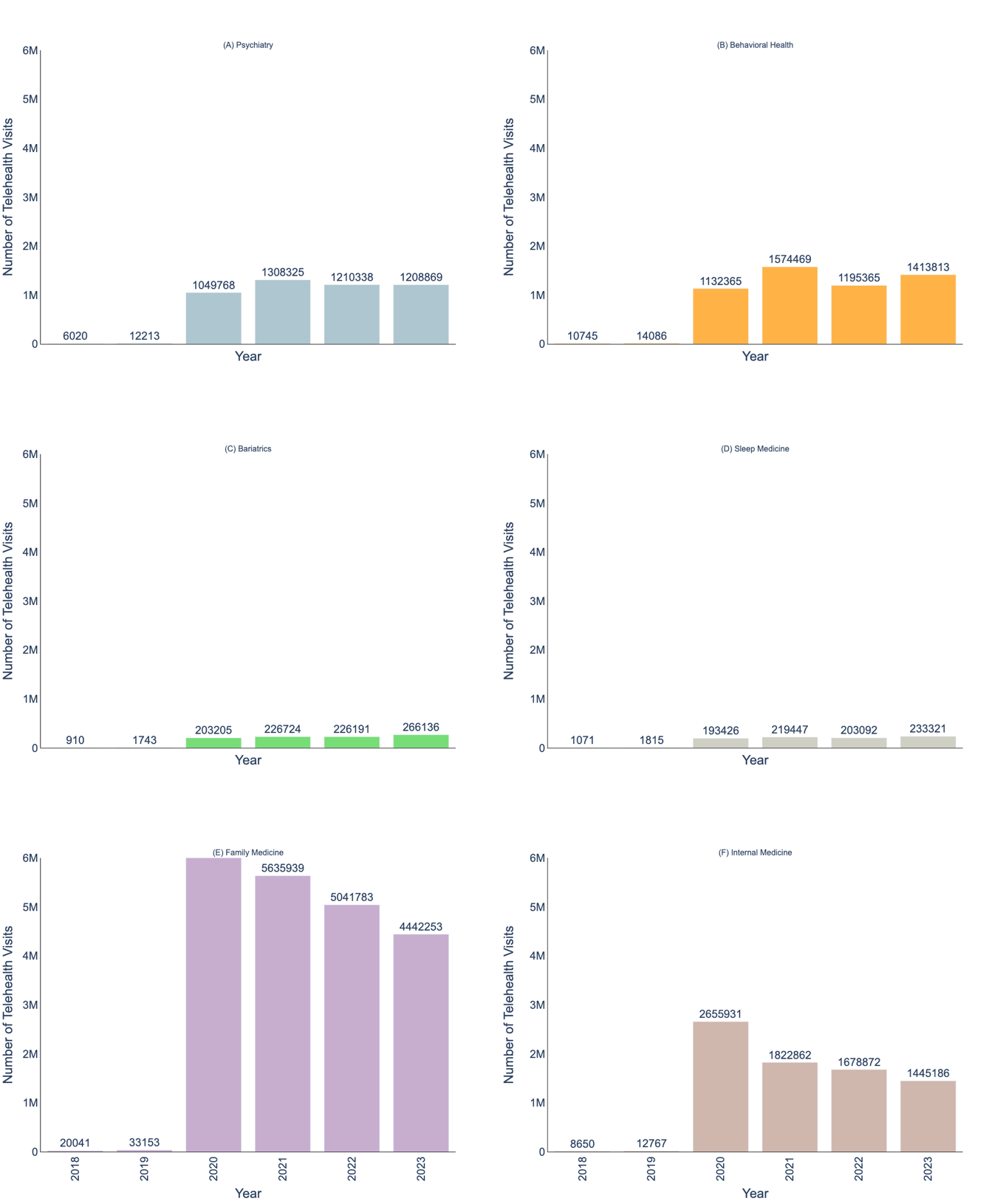


**Figure S1.** Number of telehealth visits for some specialties (2018–2023).
