# Supplementary material for: Resource Use Patterns in US Telehealth Services: Machine Learning and Clustering Analysis Across 4 Specialties
Source: JMIR Med Inform. 2026 May 7;14:e78030. doi: 10.2196/78030 (PMC13195373; doi:10.2196/78030)
Supplement: Multimedia Appendix 2 [file medinform_v14i1e78030_app2.docx]

Tables S1 and S2 show the number and percentage of visits excluded during preprocessing for each specialty.

**Table S1.** Data preprocessing for telehealth visits.

| **Preprocessing Step** | **Psychiatry** | | **Behavioral Health** | | **Bariatrics** | | **Sleep Medicine** | |
| --- | --- | --- | --- | --- | --- | --- | --- | --- |
|  | **Visits** | **Excluded** | **Visits** | **Excluded** | **Visits** | **Excluded** | **Visits** | **Excluded** |
| Initial data | 4,264,891 | - | 5,241,929 | - | 897,721 | - | 839,368 | - |
| No PatientID | 1,156 | 0.03% | 511 | 0.01% | 93 | 0.01% | 39 | 0.00% |
| No data transfer dates | 2 | 0.00% | 1 | 0.00% | 0 | 0.00% | 20 | 0.00% |
| Not within transfer window | 219,119 | 5.14% | 40,913 | 0.78% | 66,091 | 7.36% | 112,309 | 13.38% |
| Multiple healthcare providers | 11 | 0.00% | 19 | 0.00% | 4 | 0.00% | 0 | 0.00% |
| Start/end dates not the same day | 378,933 | 9.37% | 280,670 | 5.40% | 76,921 | 9.25% | 57,504 | 7.91% |
| Patients under 18 | 431,191 | 11.76% | 605,631 | 12.31% | 10,899 | 1.44% | 48,217 | 7.20% |
| Gender not specified | 1,911 | 0.06% | 4,227 | 0.10% | 166 | 0.02% | 195 | 0.03% |
| No race information | 76,956 | 2.38% | 129,509 | 3.00% | 13,600 | 1.83% | 12,929 | 2.08% |
| No RUCA code | 150 | 0.00% | 147 | 0.00% | 130 | 0.02% | 150 | 0.02% |
| No primary insurance information | 219,514 | 6.96% | 195,135 | 4.67% | 63,189 | 8.66% | 17,447 | 2.87% |
| No SVI percentiles | 7,457 | 0.25% | 8,035 | 0.27% | 1,792 | 0.27% | 1,611 | 0.27% |
| Final data | 2,929,491 | 31.33% | 3,977,131 | 24.13% | 664,836 | 25.94% | 588,947 | 29.83% |

**Table S2.** Data preprocessing for office visits.

| **Preprocessing Step** | **Psychiatry** | | **Behavioral Health** | | **Bariatrics** | | **Sleep Medicine** | |
| --- | --- | --- | --- | --- | --- | --- | --- | --- |
|  | **Visits** | **Excluded** | **Visits** | **Excluded** | **Visits** | **Excluded** | **Visits** | **Excluded** |
| Initial data | 7,331,621 | - | 16,527,421 | - | 3,355,984 | - | 4,168,268 | - |
| No PatientID | 109,550 | 1.49% | 2,870 | 0.02% | 5,033 | 0.15% | 392 | 0.01% |
| No data transfer dates | 2 | 0.00% | 22 | 0.00% | 10 | 0.00% | 0 | 0.00% |
| Not within transfer window | 237,701 | 3.29% | 122,458 | 0.74% | 149,857 | 4.47% | 217,603 | 5.22% |
| Multiple healthcare providers | 72 | 0.00% | 153 | 0.00% | 48 | 0.00% | 9 | 0.00% |
| Start/end dates not the same day | 769,562 | 11.02% | 951,646 | 5.80% | 115,929 | 3.62% | 179,472 | 4.54% |
| Patients under 18 | 757,236 | 12.18% | 2,350,627 | 15.21% | 21,819 | 0.71% | 143,515 | 3.81% |
| Gender not specified | 1,901 | 0.03% | 15,875 | 0.12% | 315 | 0.01% | 511 | 0.01% |
| No race information | 105,262 | 1.93% | 348,828 | 2.67% | 55,109 | 1.80% | 73,571 | 2.03% |
| No RUCA code | 144 | 0.00% | 143 | 0.00% | 148 | 0.00% | 138 | 0.00% |
| No primary insurance information | 407,374 | 7.61% | 1,004,596 | 7.89% | 332,714 | 11.06% | 382,288 | 10.76% |
| No SVI percentiles | 19,826 | 0.40% | 30,768 | 0.26% | 24,176 | 0.90% | 18,578 | 0.59% |
| Final data | 4,922,991 | 32.85% | 11,699,435 | 29.21% | 2,650,826 | 21.01% | 3,152,191 | 24.38% |
