# Supplementary material for: Resource Use Patterns in US Telehealth Services: Machine Learning and Clustering Analysis Across 4 Specialties
Source: JMIR Med Inform. 2026 May 7;14:e78030. doi: 10.2196/78030 (PMC13195373; doi:10.2196/78030)
Supplement: Multimedia Appendix 3 [file medinform_v14i1e78030_app3.docx]

Table S1 summarizes the predictive performance of Random Forest, XGBoost, and DNN models for patient-to-provider ratio and appointment duration across four specialties.

**Table S1.** The results of random forest, XGBoost, and DNN models.

|  | **Random Forest** | | **XGBoost** | | **DNN** | |
| --- | --- | --- | --- | --- | --- | --- |
| Specialty | **Patient-to-provider ratio** | **Appointment duration** | **Patient-to-provider ratio** | **Appointment duration** | **Patient-to-provider ratio** | **Appointment duration** |
| Psychiatry | 0.9884 | 0.6443 | 0.9935 | 0.6923 | 0.9277 | 0.6184 |
| Behavioral Health | 0.9625 | 0.6855 | 0.9689 | 0.6673 | 0.8147 | 0.5688 |
| Bariatrics | 0.9884 | 0.5985 | 0.9948 | 0.6519 | 0.9804 | 0.6571 |
| Sleep Medicine | 0.99 | 0.5475 | 0.9934 | 0.6101 | 0.9828 | 0.5294 |

For Random Forest and XGBoost, we report only the hyperparameters that influence model performance. A fixed random seed was used in all experiments to ensure reproducibility (Table S2).

**Table S2.** Key hyper parameters for Random Forest and XGBoost.

| Model | Key Hyperparameters |
| --- | --- |
| Random Forest | n_estimators = 500 |
| XGBoost | n_estimators = 500; learning_rate = 0.05; max_depth = 6; subsample = 0.8; colsample_bytree = 0.8 |

We illustrate the DNN mode configuration below in Table S3. Model architectures differed by specialty in layers, neurons, and epochs. We tested embedding dimensions, learning rates, and batch sizes. While embedding size and learning rate had little effect, smaller batch sizes improved accuracy but increased runtime. We selected a learning rate of 0.001, embedding dimension of 10, and batch size of 16 to balance performance and efficiency.

**Table S3.** DNN model configuration.

| **Specialty** | **Nr. of Hidden Layers** | **Neurons** | **Epochs** |
| --- | --- | --- | --- |
| Psychiatry | 3 | [600, 600, 400] | 100 |
| Behavioral Health | 4 | [350, 450, 650, 400] | 150 |
| Bariatrics | 3 | [550, 550, 550] | 200 |
| Sleep Medicine | 4 | [700, 800, 600, 600] | 200 |
