# Supplementary material for: Resource Use Patterns in US Telehealth Services: Machine Learning and Clustering Analysis Across 4 Specialties
Source: JMIR Med Inform. 2026 May 7;14:e78030. doi: 10.2196/78030 (PMC13195373; doi:10.2196/78030)
Supplement: Multimedia Appendix 4 [file medinform_v14i1e78030_app4.docx]

Figure S1A-D presents three clustering evaluation metrics plotted against the number of clusters for the four specialties.


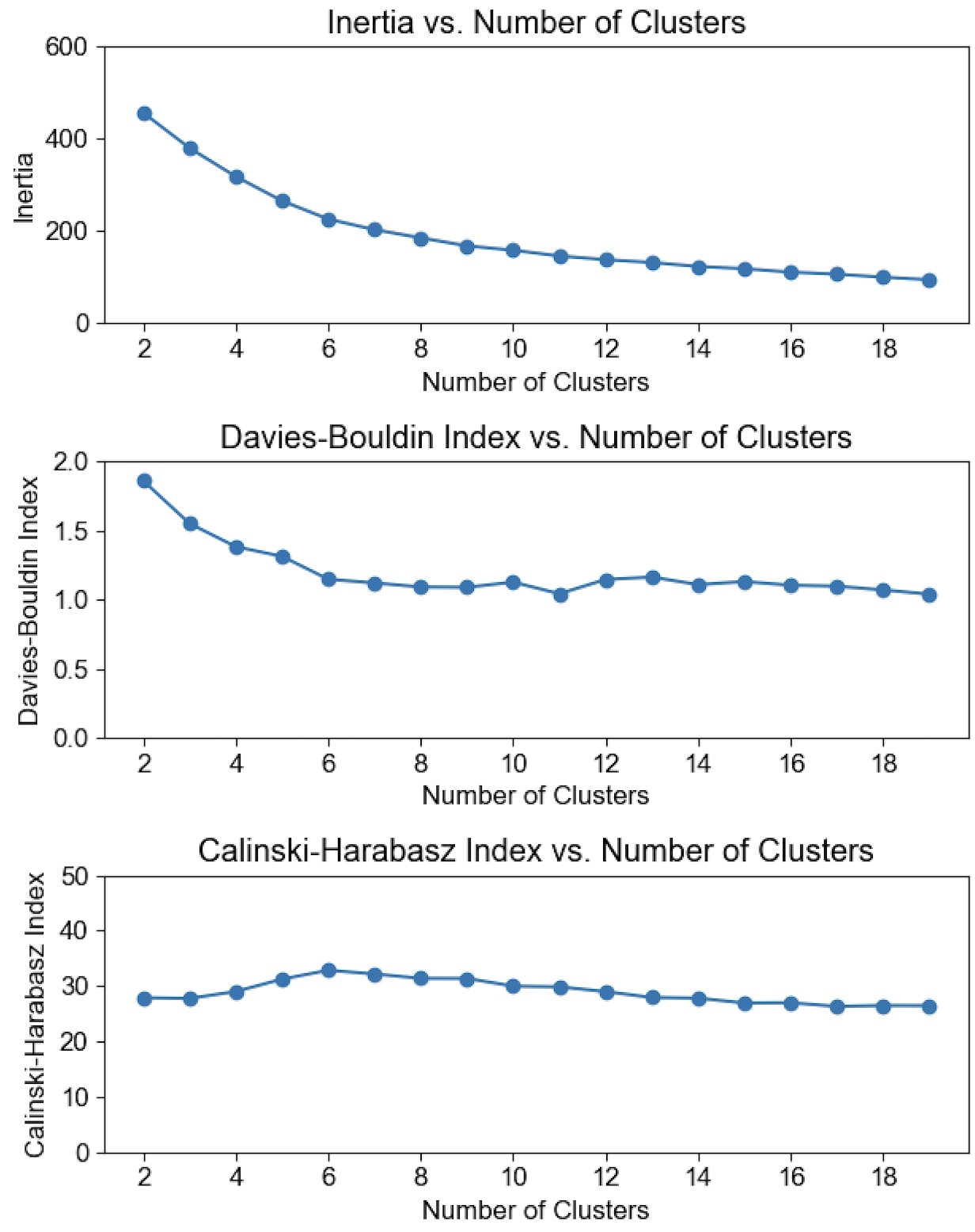

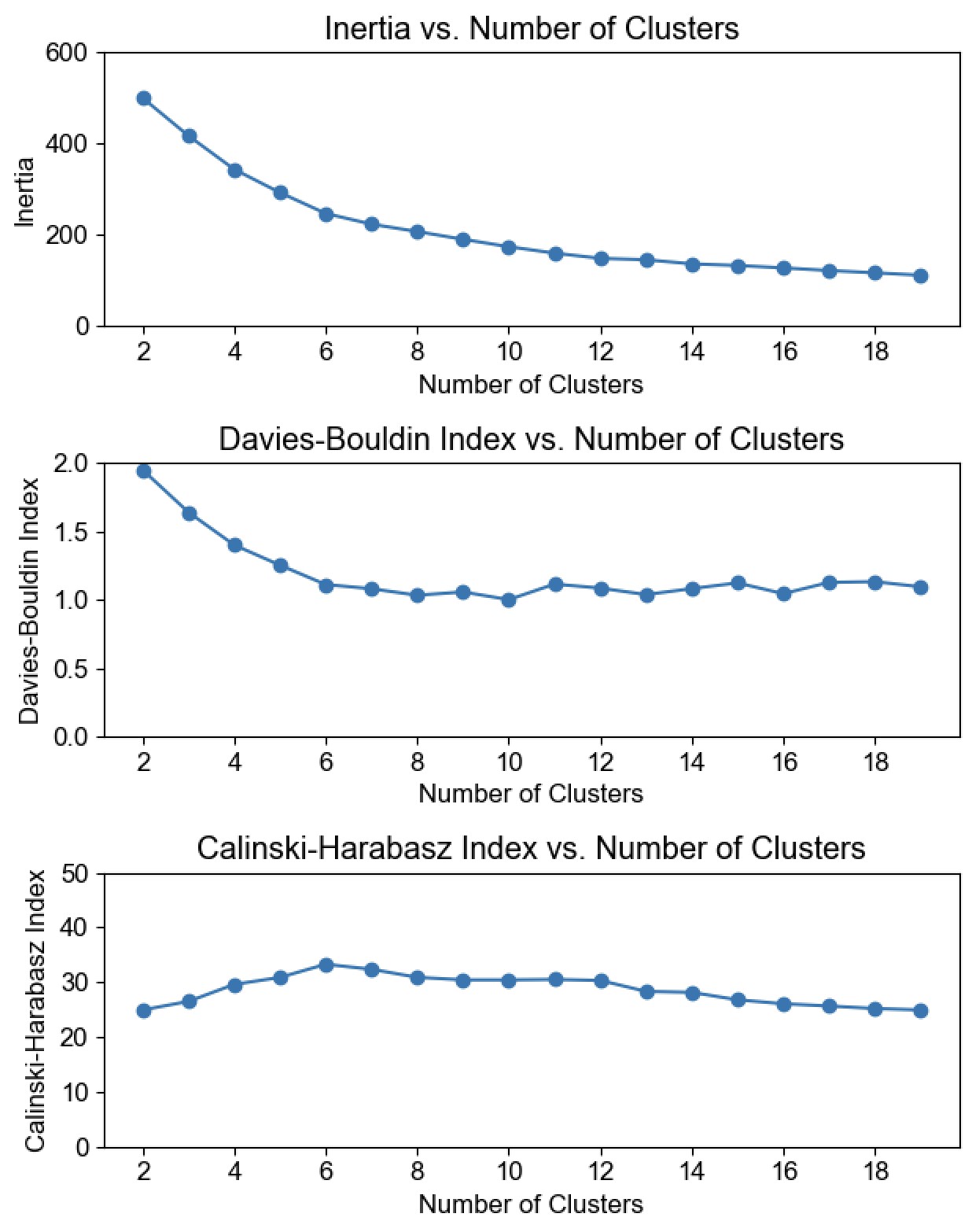


(A) Psychiatry (B) Behavioral Health


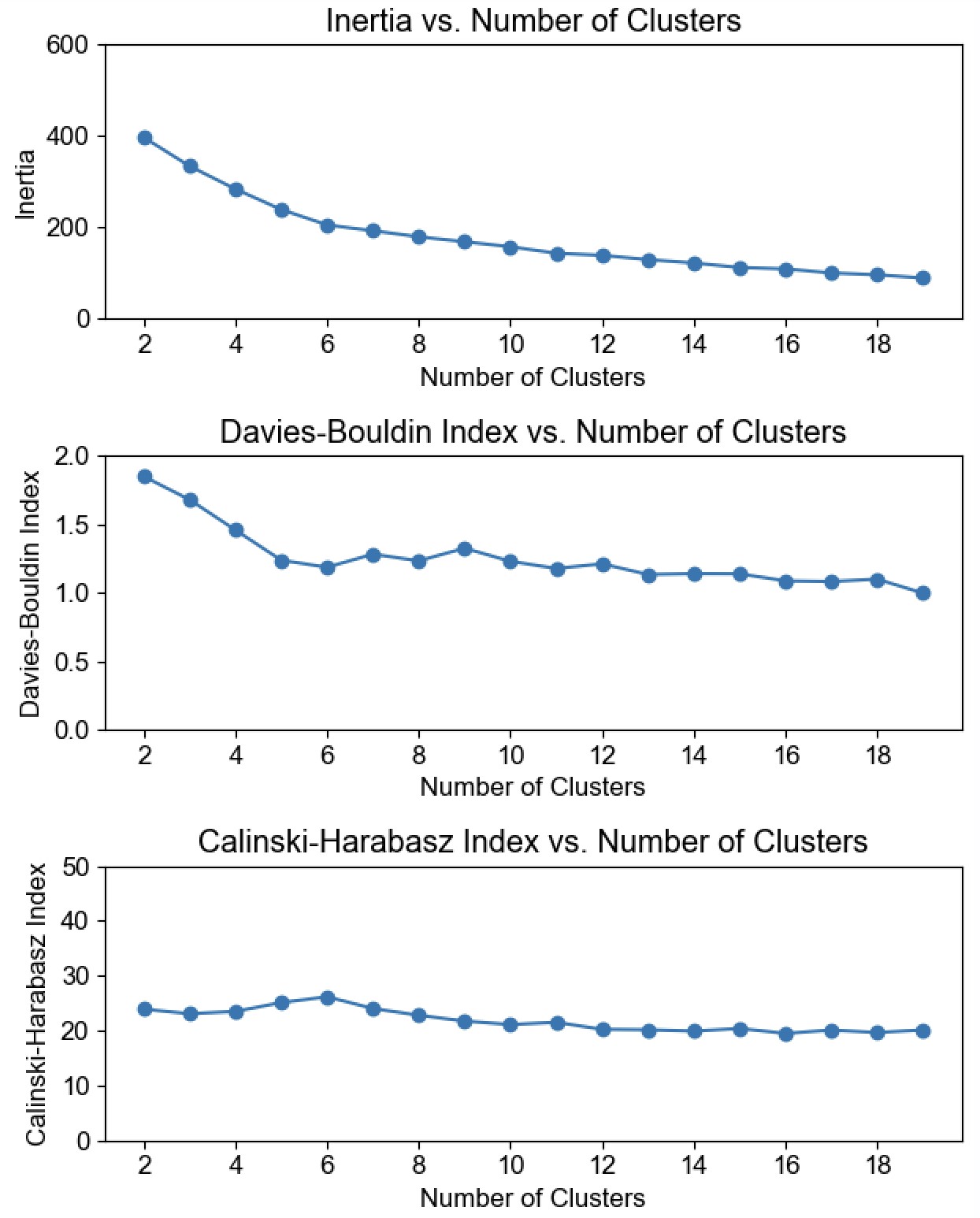

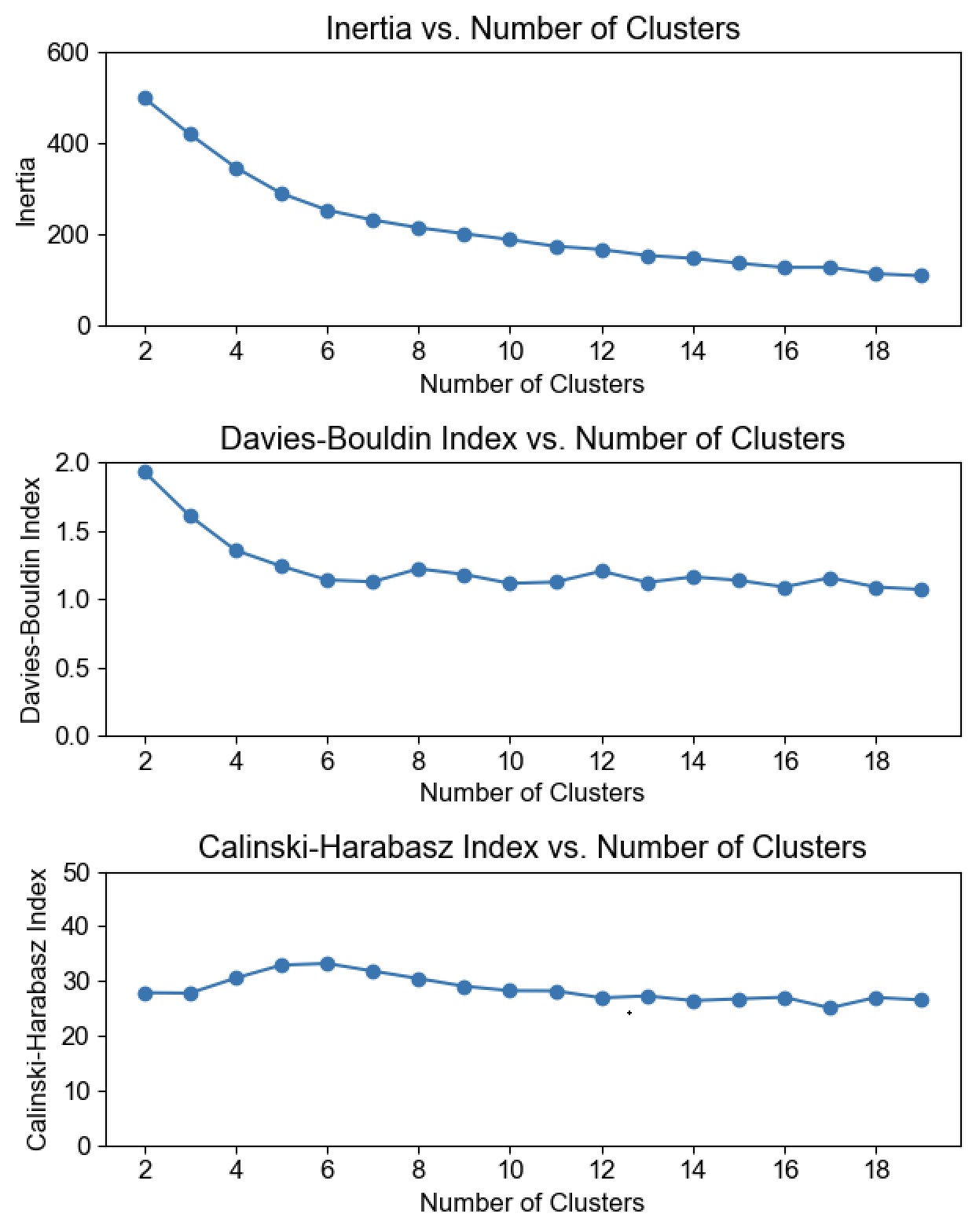


(C) Bariatrics (D) Sleep Medicine

**Figure S1.** Number of clusters vs. clustering metrics.

Table S1 presents the evaluation metrics for each specialty.

**Table S1.** Clustering metrics.

| **Specialty** | **Inertia** | **Davies-Bouldin Index** | **Calinski-Harabasz Index** |
| --- | --- | --- | --- |
| Psychiatry | 226.13 | 1.15 | 32.85 |
| Behavioral Health | 246.98 | 1.11 | 33.34 |
| Bariatrics | 205.63 | 1.18 | 26.17 |
| Sleep Medicine | 253.86 | 1.12 | 33.29 |
