# Supplementary material for: Resource Use Patterns in US Telehealth Services: Machine Learning and Clustering Analysis Across 4 Specialties
Source: JMIR Med Inform. 2026 May 7;14:e78030. doi: 10.2196/78030 (PMC13195373; doi:10.2196/78030)
Supplement: Multimedia Appendix 5 [file medinform_v14i1e78030_app5.docx]

Figure S1A-D illustrates the importance of features in the clustering model across the four specialties.


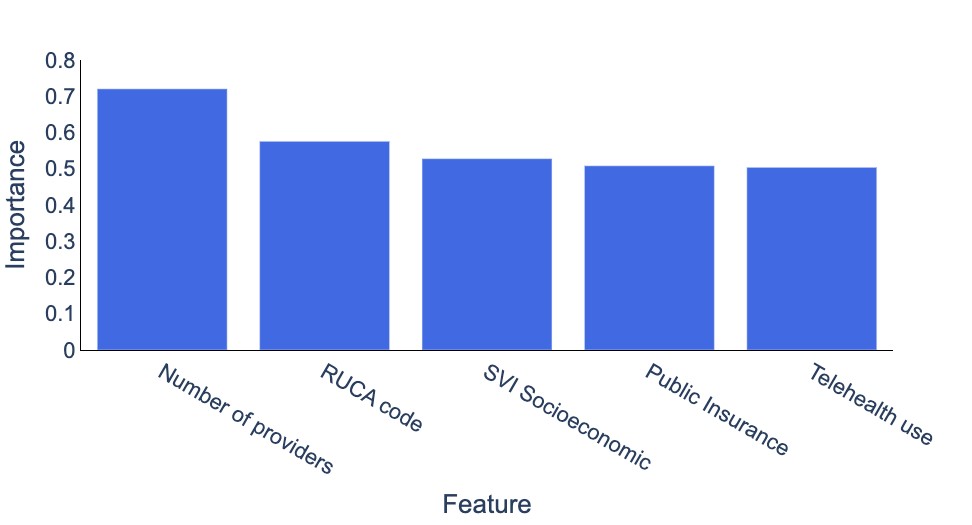

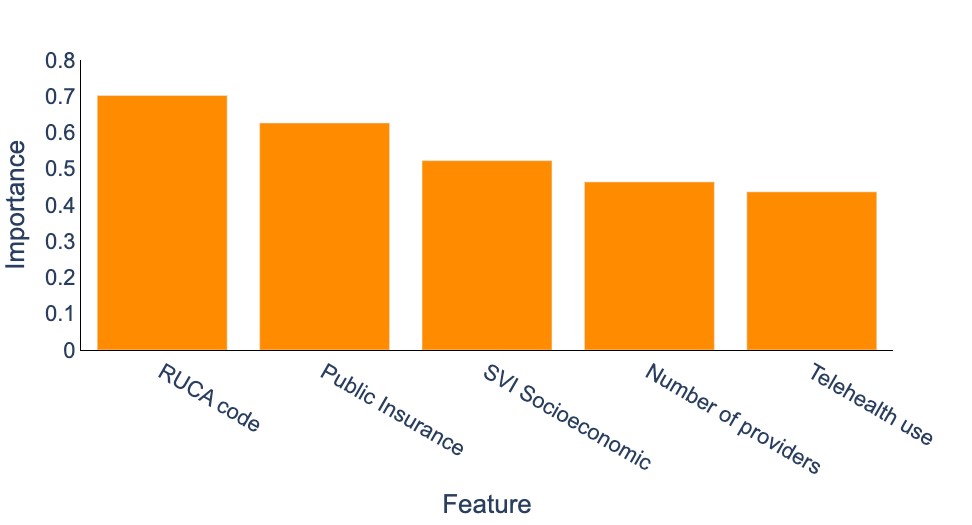


(A) Psychiatry (B) Behavioral Health


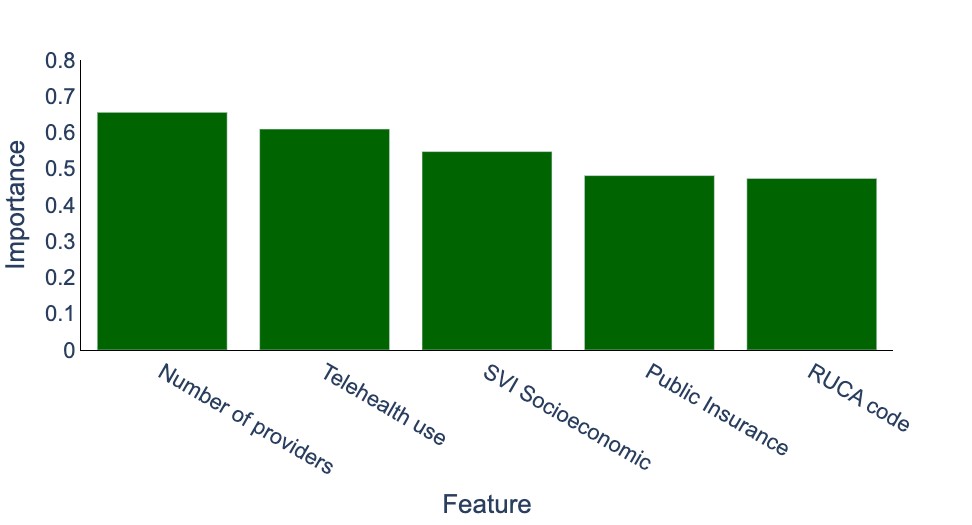

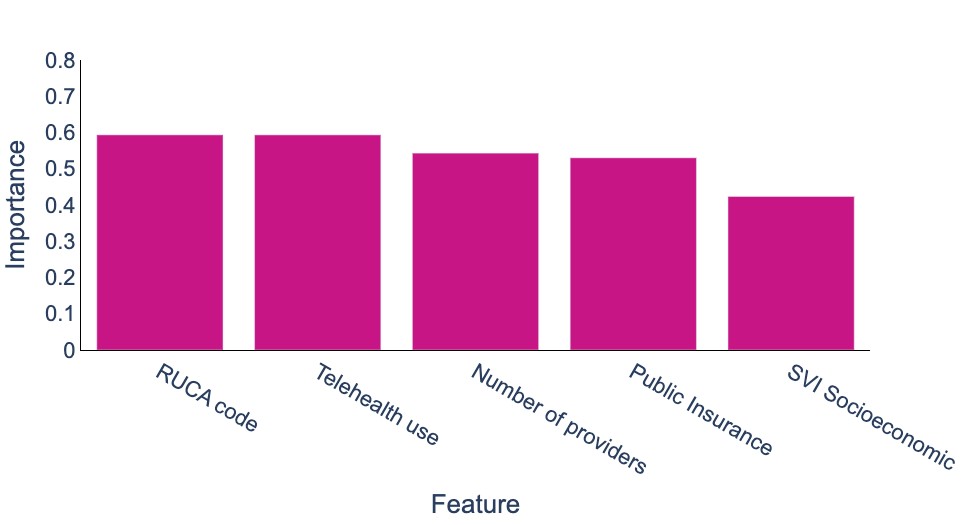


(C) Bariatrics (D) Sleep Medicine

**Figure S1.** Feature importance for clustering models.
