# Supplementary material for: Resource Use Patterns in US Telehealth Services: Machine Learning and Clustering Analysis Across 4 Specialties
Source: JMIR Med Inform. 2026 May 7;14:e78030. doi: 10.2196/78030 (PMC13195373; doi:10.2196/78030)
Supplement: Multimedia Appendix 6 [file medinform_v14i1e78030_app6.docx]

Tables S1-S4 display the number of facilities, average feature values for each cluster, and the average patient-to-provider ratios and appointment durations across clusters for each specialty.

**Table S1.** Psychiatry - Summary results of the clustering model.

| **Clusters** | **Nr. of facilities** | **Age** | **RUCA code** | **SVI Household** | **SVI Housing** | **SVI Minority** | **SVI Socioeconomic** |
| --- | --- | --- | --- | --- | --- | --- | --- |
| Cluster 1 | 19 | 40.68 | 1.39 | 0.38 | 0.55 | 0.62 | 0.35 |
| Cluster 2 | 10 | 49.46 | 3.59 | 0.38 | 0.65 | 0.49 | 0.39 |
| Cluster 3 | 21 | 49.89 | 1.68 | 0.49 | 0.63 | 0.64 | 0.5 |
| Cluster 4 | 23 | 43.89 | 1.28 | 0.41 | 0.6 | 0.72 | 0.46 |
| Cluster 5 | 22 | 45.63 | 1.63 | 0.61 | 0.67 | 0.71 | 0.61 |
| Cluster 6 | 19 | 46.54 | 1.27 | 0.31 | 0.53 | 0.68 | 0.35 |
| **Clusters** | **Nr. Of facilities** | **Race** | **Gender** | **Public Insurance** | **Private Insurance** | **Self-pay** | **Rurality** |
| Cluster 1 | 19 | 0.88 | 0.6 | 0.22 | 0.77 | 0.01 | 0.04 |
| Cluster 2 | 10 | 0.94 | 0.68 | 0.3 | 0.69 | 0.01 | 0.19 |
| Cluster 3 | 21 | 0.79 | 0.64 | 0.55 | 0.44 | 0.02 | 0.07 |
| Cluster 4 | 23 | 0.78 | 0.67 | 0.25 | 0.72 | 0.03 | 0.03 |
| Cluster 5 | 22 | 0.75 | 0.71 | 0.19 | 0.79 | 0.01 | 0.06 |
| Cluster 6 | 19 | 0.85 | 0.75 | 0.13 | 0.82 | 0.06 | 0.04 |
| **Clusters** | **Nr. of facilities** | **Total visits** | **Telehealth visits** | **Nr. of providers** | **Telehealth use** | **Appointment duration** | **Patient/provider ratio** |
| Cluster 1 | 19 | 32930 | 7082 | 46 | 0.11 | 38.7 | 26.86 |
| Cluster 2 | 10 | 22200 | 5647 | 35 | 0.27 | 40.07 | 25.65 |
| Cluster 3 | 21 | 26284 | 7866 | 64 | 0.36 | 40.57 | 14.78 |
| Cluster 4 | 23 | 133198 | 48585 | 290 | 0.43 | 47.63 | 13.99 |
| Cluster 5 | 22 | 31722 | 7691 | 40 | 0.25 | 42.25 | 27.67 |
| Cluster 6 | 19 | 11872 | 10607 | 31 | 0.88 | 32.32 | 11.26 |

**Table S2.** Behavioral Health - Summary results of the clustering model.

| **Clusters** | **Nr. of facilities** | **Age** | **RUCA code** | **SVI Household** | **SVI Housing** | **SVI Minority** | **SVI Socioeconomic** |
| --- | --- | --- | --- | --- | --- | --- | --- |
| Cluster 1 | 21 | 41.2 | 1.56 | 0.51 | 0.64 | 0.71 | 0.54 |
| Cluster 2 | 36 | 42.11 | 1.23 | 0.41 | 0.57 | 0.68 | 0.41 |
| Cluster 3 | 17 | 43.77 | 1.43 | 0.31 | 0.52 | 0.66 | 0.32 |
| Cluster 4 | 13 | 42.33 | 1.52 | 0.37 | 0.58 | 0.62 | 0.42 |
| Cluster 5 | 19 | 43.1 | 3.33 | 0.58 | 0.65 | 0.49 | 0.51 |
| Cluster 6 | 15 | 39.95 | 1.16 | 0.55 | 0.75 | 0.78 | 0.72 |
| **Clusters** | **Nr. Of facilities** | **Race** | **Gender** | **Public Insurance** | **Private Insurance** | **Self-pay** | **Rurality** |
| Cluster 1 | 21 | 0.77 | 0.66 | 0.57 | 0.42 | 0.01 | 0.06 |
| Cluster 2 | 36 | 0.77 | 0.63 | 0.23 | 0.76 | 0.01 | 0.03 |
| Cluster 3 | 17 | 0.85 | 0.76 | 0.13 | 0.75 | 0.13 | 0.05 |
| Cluster 4 | 13 | 0.86 | 0.67 | 0.14 | 0.86 | 0.01 | 0.06 |
| Cluster 5 | 19 | 0.89 | 0.71 | 0.27 | 0.73 | 0.01 | 0.21 |
| Cluster 6 | 15 | 0.64 | 0.59 | 0.09 | 0.89 | 0.02 | 0.02 |
| **Clusters** | **Nr. of facilities** | **Total visits** | **Telehealth visits** | **Nr. of providers** | **Telehealth use** | **Appointment duration** | **Patient/provider ratio** |
| Cluster 1 | 21 | 33649 | 19526 | 87 | 0.61 | 45.81 | 13.49 |
| Cluster 2 | 36 | 21856 | 1813 | 40 | 0.09 | 46.75 | 16.05 |
| Cluster 3 | 17 | 16975 | 12870 | 40 | 0.83 | 47.61 | 14.09 |
| Cluster 4 | 13 | 129096 | 39890 | 288 | 0.23 | 46.29 | 15.22 |
| Cluster 5 | 19 | 82519 | 18533 | 89 | 0.2 | 46.73 | 19.52 |
| Cluster 6 | 15 | 7498 | 3544 | 33 | 0.55 | 34.17 | 9.16 |

**Table S3.** Bariatrics - Summary results of the clustering model.

| **Clusters** | **Nr. of facilities** | **Age** | **RUCA code** | **SVI Household** | **SVI Housing** | **SVI Minority** | **SVI Socioeconomic** |
| --- | --- | --- | --- | --- | --- | --- | --- |
| Cluster 1 | 11 | 50.03 | 3.34 | 0.52 | 0.61 | 0.42 | 0.39 |
| Cluster 2 | 20 | 47.97 | 1.29 | 0.46 | 0.61 | 0.74 | 0.52 |
| Cluster 3 | 21 | 50.91 | 1.42 | 0.38 | 0.55 | 0.69 | 0.37 |
| Cluster 4 | 10 | 42.03 | 1.72 | 0.55 | 0.61 | 0.68 | 0.56 |
| Cluster 5 | 19 | 49.96 | 1.73 | 0.5 | 0.58 | 0.62 | 0.49 |
| Cluster 6 | 18 | 48.64 | 1.72 | 0.57 | 0.64 | 0.71 | 0.62 |
| **Clusters** | **Nr. Of facilities** | **Race** | **Gender** | **Public Insurance** | **Private Insurance** | **Self-pay** | **Rurality** |
| Cluster 1 | 11 | 0.93 | 0.8 | 0.26 | 0.74 | 0 | 0.26 |
| Cluster 2 | 20 | 0.62 | 0.82 | 0.14 | 0.85 | 0.01 | 0.01 |
| Cluster 3 | 21 | 0.69 | 0.8 | 0.13 | 0.85 | 0.02 | 0.04 |
| Cluster 4 | 10 | 0.66 | 0.8 | 0.48 | 0.52 | 0 | 0.07 |
| Cluster 5 | 19 | 0.76 | 0.8 | 0.27 | 0.72 | 0.01 | 0.05 |
| Cluster 6 | 18 | 0.59 | 0.82 | 0.1 | 0.89 | 0.01 | 0.05 |
| **Clusters** | **Nr. of facilities** | **Total visits** | **Telehealth visits** | **Nr. of providers** | **Telehealth use** | **Appointment duration** | **Patient/provider ratio** |
| Cluster 1 | 11 | 20569 | 2047 | 21 | 0.07 | 40.82 | 25.64 |
| Cluster 2 | 20 | 25156 | 9888 | 34 | 0.42 | 40.88 | 29.13 |
| Cluster 3 | 21 | 19242 | 3895 | 21 | 0.15 | 39.77 | 28.77 |
| Cluster 4 | 10 | 9418 | 1057 | 18 | 0.13 | 45.3 | 21.64 |
| Cluster 5 | 19 | 58345 | 6925 | 71 | 0.11 | 42.7 | 30.19 |
| Cluster 6 | 18 | 16321 | 793 | 18 | 0.05 | 47.09 | 42.22 |

**Table S4.** Sleep Medicine - Summary results of the clustering model.

| **Clusters** | **Nr. of facilities** | **Age** | **RUCA code** | **SVI Household** | **SVI Housing** | **SVI Minority** | **SVI Socioeconomic** |
| --- | --- | --- | --- | --- | --- | --- | --- |
| Cluster 1 | 15 | 53.63 | 1.73 | 0.6 | 0.68 | 0.73 | 0.66 |
| Cluster 2 | 34 | 58.51 | 1.4 | 0.38 | 0.53 | 0.65 | 0.36 |
| Cluster 3 | 27 | 55.65 | 1.59 | 0.53 | 0.57 | 0.67 | 0.51 |
| Cluster 4 | 17 | 62.29 | 3.32 | 0.57 | 0.61 | 0.48 | 0.49 |
| Cluster 5 | 12 | 60.38 | 1.64 | 0.45 | 0.55 | 0.59 | 0.43 |
| Cluster 6 | 18 | 61.29 | 1.31 | 0.34 | 0.58 | 0.71 | 0.39 |
| **Clusters** | **Nr. Of facilities** | **Race** | **Gender** | **Public Insurance** | **Private Insurance** | **Self-pay** | **Rurality** |
| Cluster 1 | 15 | 0.68 | 0.5 | 0.19 | 0.79 | 0.02 | 0.07 |
| Cluster 2 | 34 | 0.8 | 0.46 | 0.23 | 0.77 | 0 | 0.04 |
| Cluster 3 | 27 | 0.76 | 0.51 | 0.5 | 0.49 | 0 | 0.04 |
| Cluster 4 | 17 | 0.92 | 0.47 | 0.35 | 0.65 | 0 | 0.21 |
| Cluster 5 | 12 | 0.8 | 0.52 | 0.23 | 0.74 | 0.03 | 0.07 |
| Cluster 6 | 18 | 0.8 | 0.47 | 0.3 | 0.7 | 0 | 0.03 |
| **Clusters** | **Nr. of facilities** | **Total visits** | **Telehealth visits** | **Nr. of providers** | **Telehealth use** | **Appointment duration** | **Patient/provider ratio** |
| Cluster 1 | 15 | 10651 | 1482 | 10 | 0.05 | 33.39 | 29.29 |
| Cluster 2 | 34 | 16333 | 1747 | 16 | 0.07 | 36.31 | 33.12 |
| Cluster 3 | 27 | 18607 | 1083 | 19 | 0.06 | 39.55 | 33.39 |
| Cluster 4 | 17 | 30277 | 3737 | 23 | 0.15 | 32.28 | 44.66 |
| Cluster 5 | 12 | 88162 | 9171 | 104 | 0.09 | 40.88 | 41.45 |
| Cluster 6 | 18 | 32594 | 13282 | 30 | 0.47 | 33.84 | 34.15 |


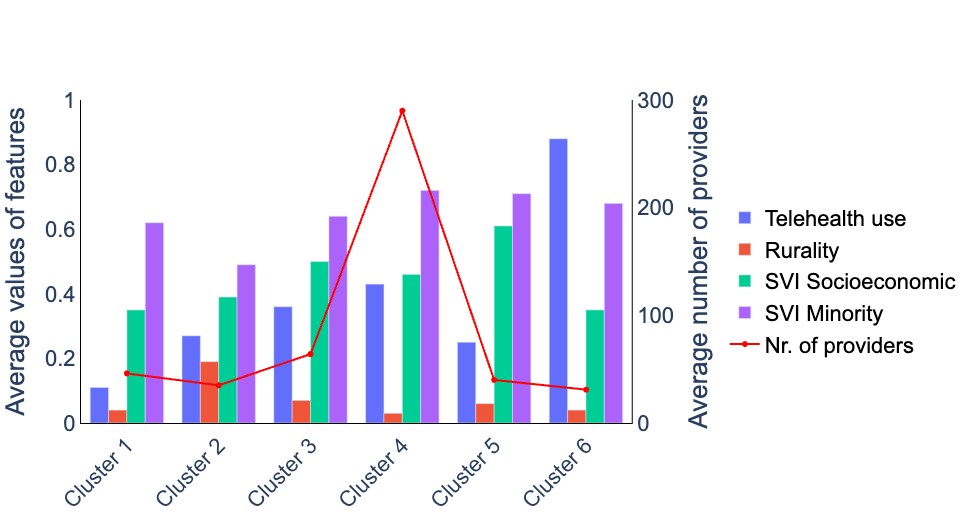

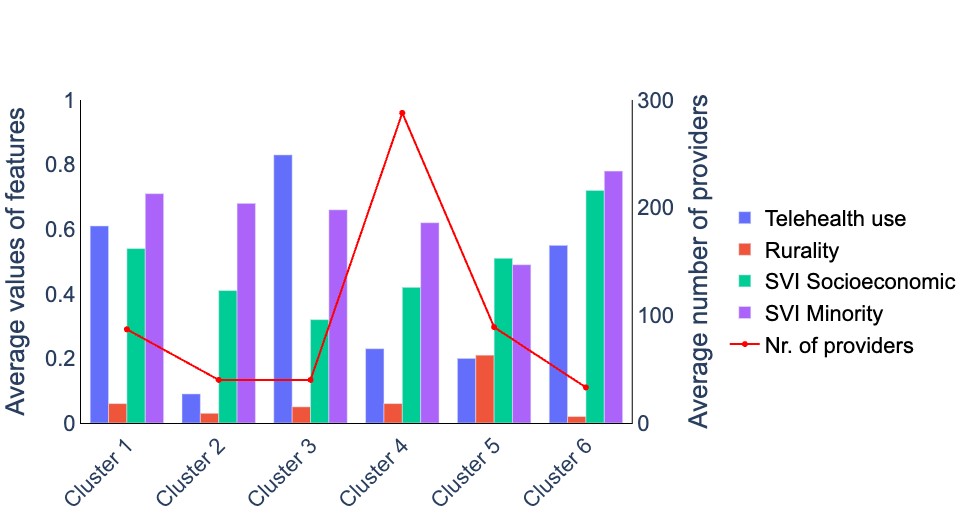


(A) Psychiatry (B) Behavioral Health


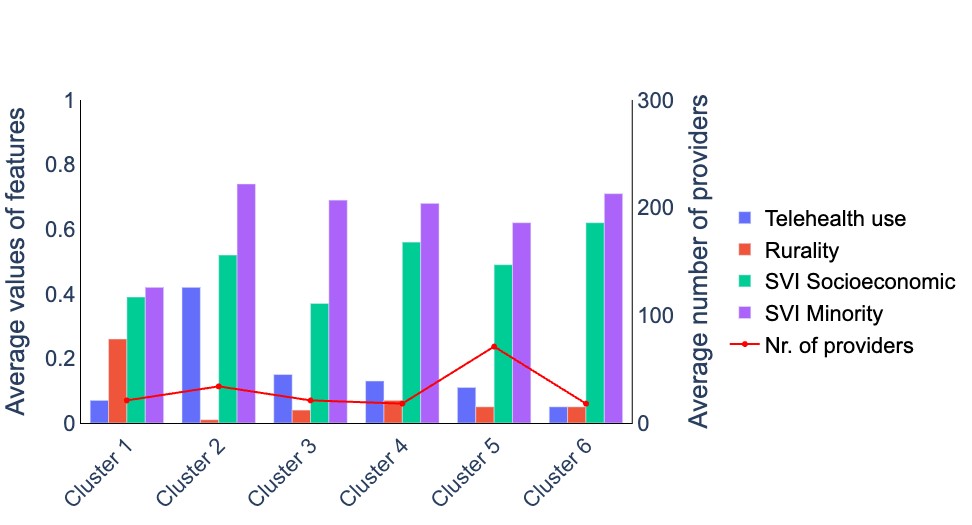

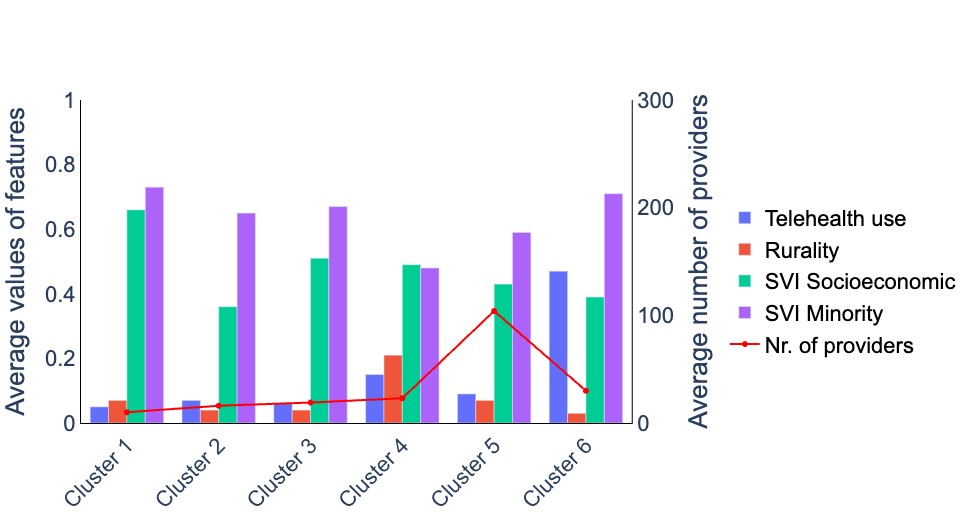


(C) Bariatrics (D) Sleep Medicine

**Figure S1.** Features and providers across clusters.


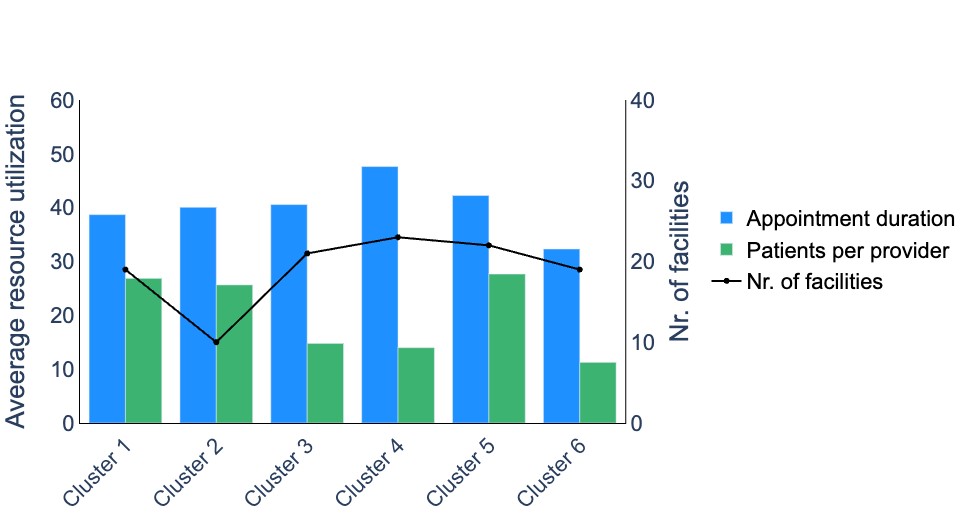

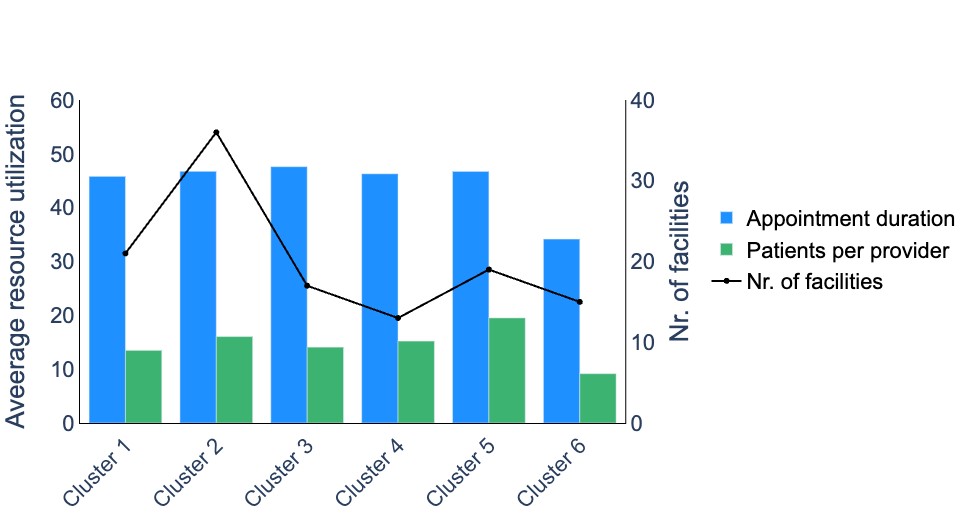


(A) Psychiatry (B) Behavioral Health


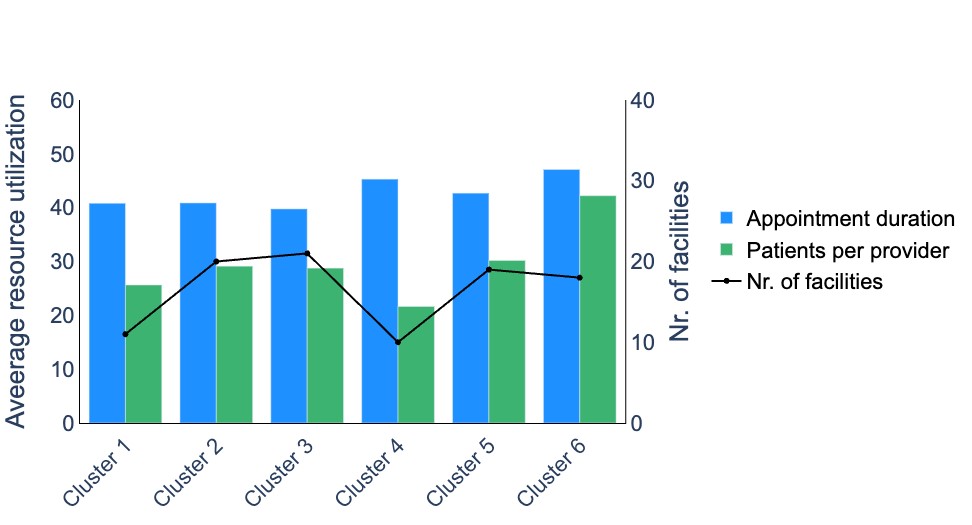

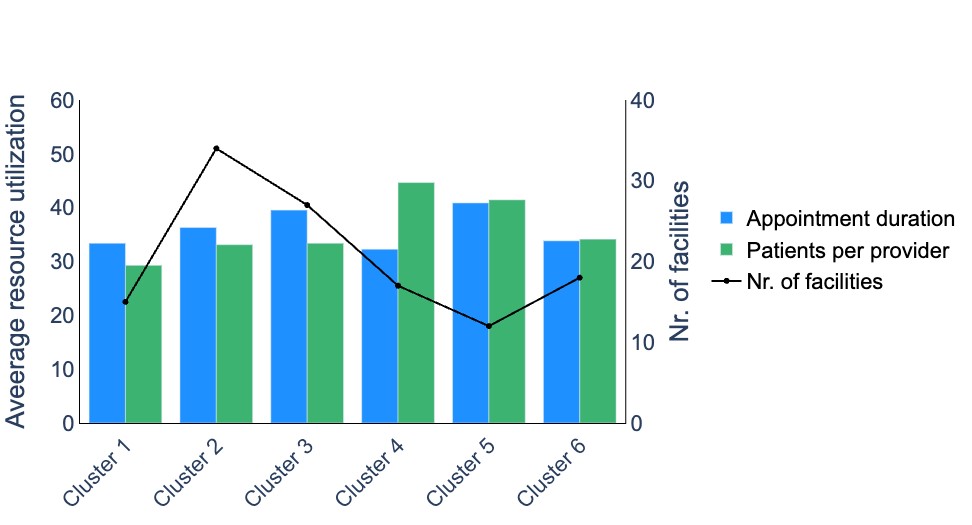


(C) Bariatrics (D) Sleep Medicine

**Figure S2.** Patient-to-provider ratios and appointment durations across clusters.
