# Supplementary material for: Resource Use Patterns in US Telehealth Services: Machine Learning and Clustering Analysis Across 4 Specialties
Source: JMIR Med Inform. 2026 May 7;14:e78030. doi: 10.2196/78030 (PMC13195373; doi:10.2196/78030)
Supplement: Multimedia Appendix 7 [file medinform_v14i1e78030_app7.docx]

Table S1 presents the average patient-to-provider ratios for telehealth and office visits during and post-pandemic, segmented by clusters, while Table S2 provides the corresponding appointment duration data.

**Table S1.** Average patient-to-provider ratios across the clusters.

| **Specialty** | **Cluster** | **Telehealth** | | **Office** | |
| --- | --- | --- | --- | --- | --- |
|  |  | **During-pandemic** | **Post-pandemic** | **During-pandemic** | **Post-pandemic** |
| Psychiatry | 1 | 16.81 | 28.98 | 15.83 | 34.88 |
|  | 2 | 14.14 | 33.20 | 26.72 | 31.12 |
|  | 3 | 16.20 | 15.31 | 13.73 | 23.51 |
|  | 4 | 11.75 | 10.54 | 9.86 | 15.24 |
|  | 5 | 18.27 | 40.36 | 20.01 | 41.10 |
|  | 6 | 28.72 | 20.06 | 20.71 | 11.61 |
| Behavioral Health | 1 | 15.67 | 12.10 | 13.76 | 15.75 |
|  | 2 | 12.78 | 18.37 | 10.97 | 19.48 |
|  | 3 | 21.11 | 9.06 | 18.38 | 16.75 |
|  | 4 | 12.01 | 15.96 | 11.02 | 17.51 |
|  | 5 | 14.55 | 20.39 | 14.33 | 25.82 |
|  | 6 | 12.97 | 13.83 | 15.11 | 20.55 |
| Bariatrics | 1 | 21.04 | 29.58 | 10.64 | 39.82 |
|  | 2 | 25.33 | 22.45 | 30.62 | 34.24 |
|  | 3 | 22.82 | 34.21 | 23.48 | 40.39 |
|  | 4 | 6.40 | 17.70 | 11.72 | 30.62 |
|  | 5 | 14.18 | 31.59 | 13.97 | 37.83 |
|  | 6 | 8.82 | 40.55 | 6.62 | 46.77 |
| Sleep Medicine | 1 | 36.31 | 50.63 | 25.95 | 58.21 |
|  | 2 | 18.48 | 40.70 | 13.56 | 51.90 |
|  | 3 | 15.53 | 44.07 | 9.99 | 51.69 |
|  | 4 | 21.85 | 52.34 | 20.76 | 66.59 |
|  | 5 | 18.25 | 47.92 | 17.33 | 53.94 |
|  | 6 | 37.66 | 26.29 | 38.46 | 41.10 |

**Table S2.** Average appointment durations (mins) across the clusters.

| **Specialty** | **Cluster** | **Telehealth** | | **Office** | |
| --- | --- | --- | --- | --- | --- |
|  |  | **During-pandemic** | **Post-pandemic** | **During-pandemic** | **Post-pandemic** |
| Psychiatry | 1 | 39.85 | 38.08 | 44.33 | 44.28 |
|  | 2 | 34.97 | 32.78 | 43.67 | 43.96 |
|  | 3 | 41.60 | 40.39 | 39.79 | 42.09 |
|  | 4 | 47.14 | 44.82 | 51.25 | 50.16 |
|  | 5 | 40.79 | 39.04 | 44.20 | 44.05 |
|  | 6 | 45.38 | 40.23 | 55.26 | 53.56 |
| Behavioral Health | 1 | 50.33 | 45.46 | 52.41 | 48.86 |
|  | 2 | 51.06 | 50.33 | 49.79 | 48.50 |
|  | 3 | 52.63 | 48.78 | 57.03 | 53.93 |
|  | 4 | 43.49 | 40.85 | 49.09 | 47.13 |
|  | 5 | 41.61 | 41.85 | 47.90 | 48.50 |
|  | 6 | 49.26 | 44.83 | 48.05 | 46.76 |
| Bariatrics | 1 | 45.51 | 33.02 | 48.93 | 46.20 |
|  | 2 | 34.85 | 35.39 | 43.4 | 44.69 |
|  | 3 | 34.30 | 31.20 | 40.43 | 40.62 |
|  | 4 | 38.10 | 29.52 | 44.87 | 47.47 |
|  | 5 | 39.51 | 35.00 | 44.65 | 43.43 |
|  | 6 | 44.61 | 34.73 | 49.94 | 48.91 |
| Sleep Medicine | 1 | 42.15 | 45.39 | 46.29 | 44.12 |
|  | 2 | 35.06 | 28.43 | 40.77 | 41.09 |
|  | 3 | 37.83 | 31.60 | 42.06 | 42.05 |
|  | 4 | 32.63 | 25.01 | 35.15 | 34.96 |
|  | 5 | 34.12 | 33.89 | 41.07 | 42.39 |
|  | 6 | 30.79 | 27.52 | 36.92 | 37.46 |


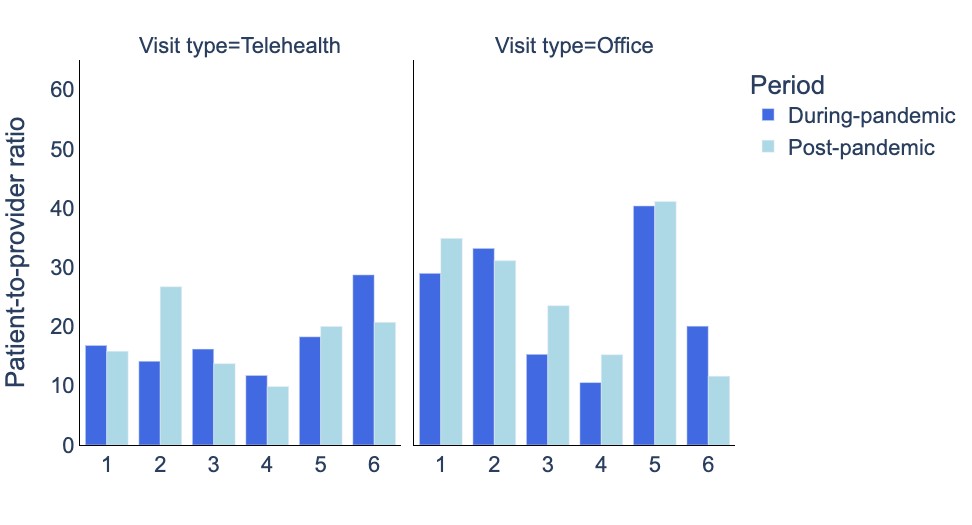

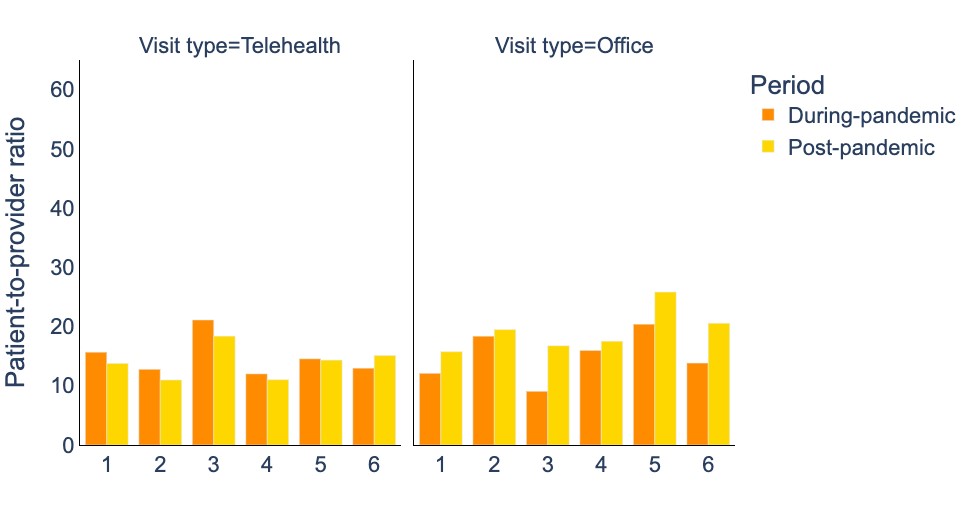


(A) Psychiatry (B) Behavioral Health


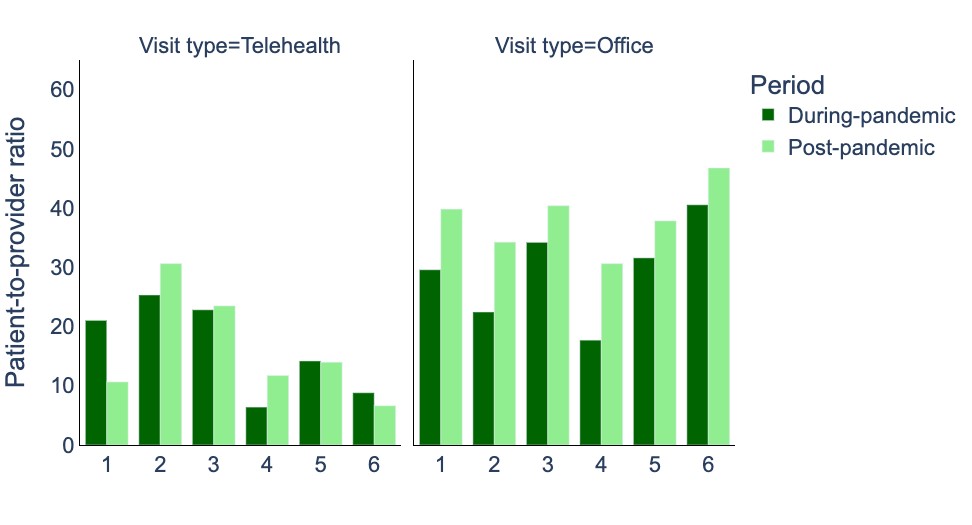

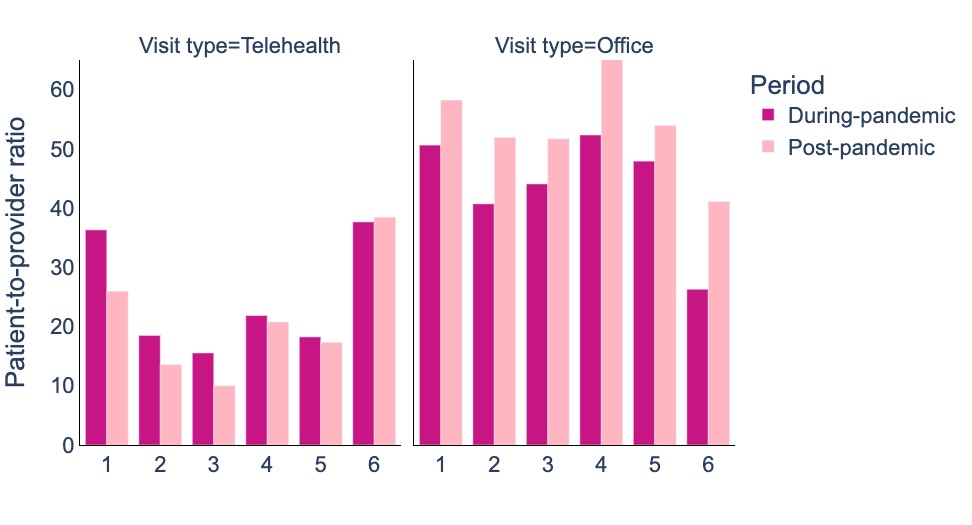


(C) Bariatrics (D) Sleep Medicine

**Figure S1.** Patient-to-provider ratio across the clusters for the selected specialties.


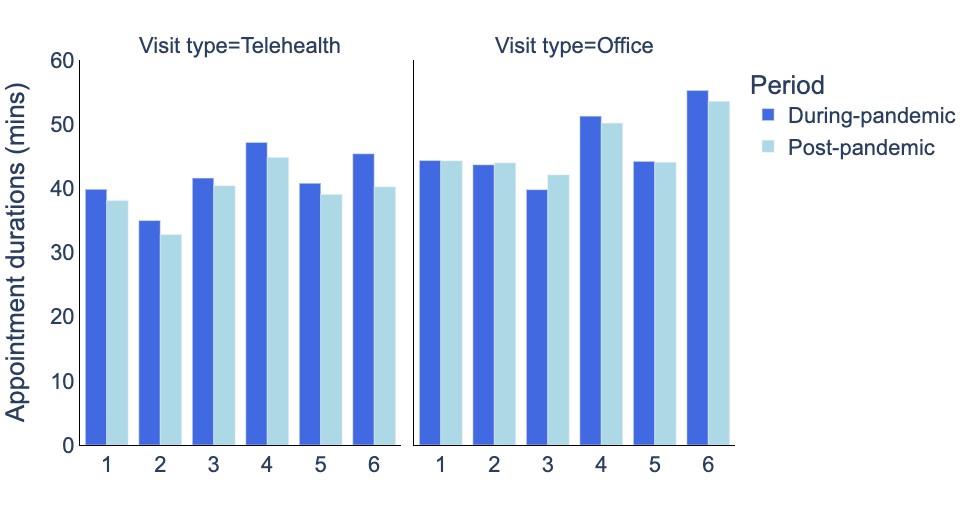

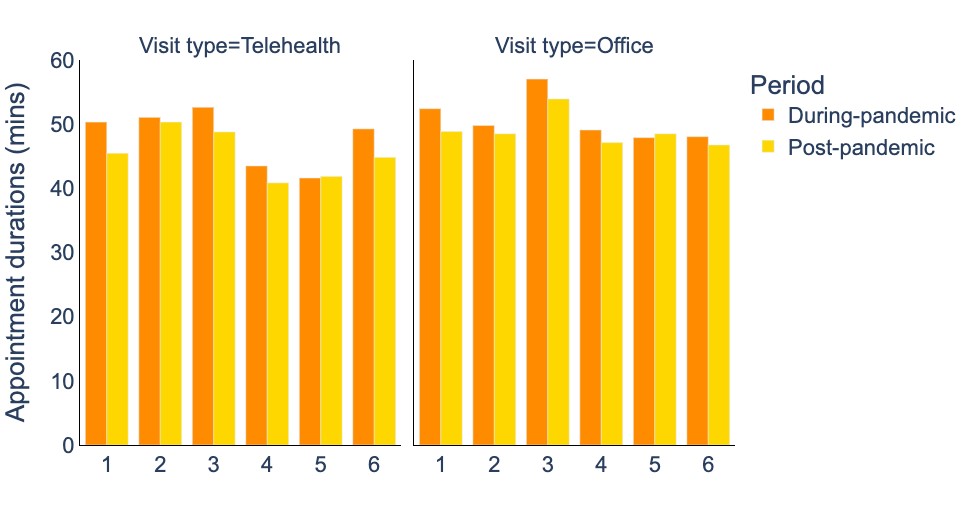


(A) Psychiatry (B) Behavioral Health


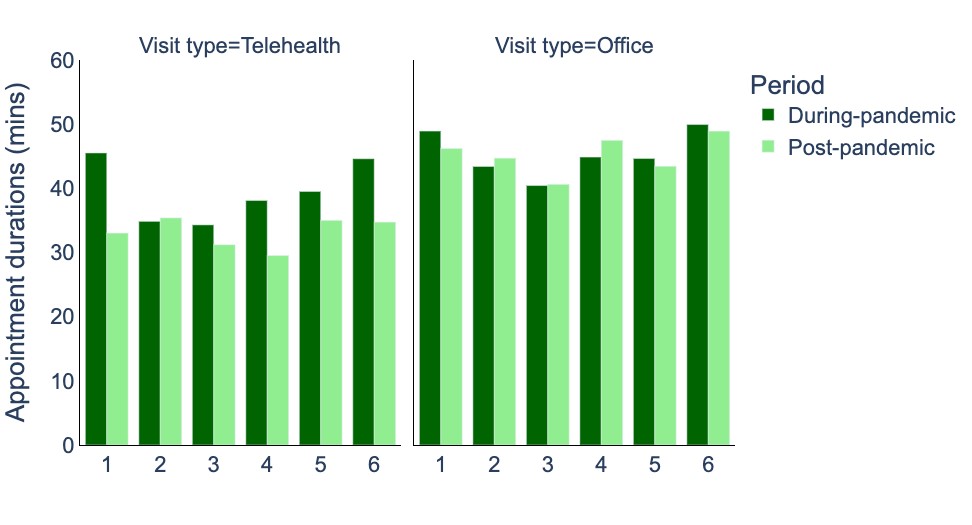

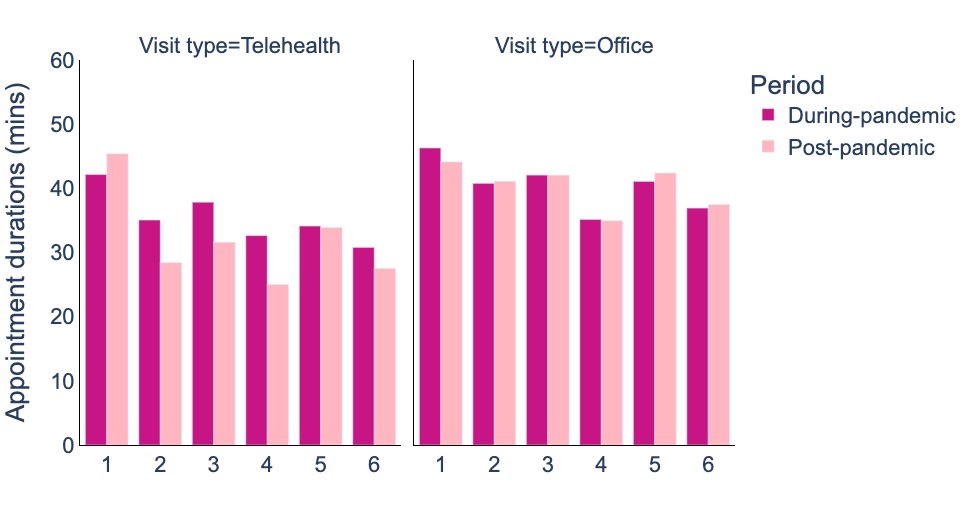


(C) Bariatrics (D) Sleep Medicine

**Figure S2.** Appointment duration (mins) across the clusters for the selected specialties.
