# Supplementary material for: Resource Use Patterns in US Telehealth Services: Machine Learning and Clustering Analysis Across 4 Specialties
Source: JMIR Med Inform. 2026 May 7;14:e78030. doi: 10.2196/78030 (PMC13195373; doi:10.2196/78030)
Supplement: Multimedia Appendix 8 [file medinform_v14i1e78030_app8.docx]

Tables S1-S8 present the results of Dunn’s test with corresponding *P-*values for pairwise comparisons across clusters. The *P*-values below the significance threshold (α=.05) indicate statistically significant differences between clusters.

**Table S1.** Dunn’s test results for Psychiatry - patient-to-provider ratios.

|  | **Telehealth, during-pandemic** | | | | | **Office, during-pandemic** | | | | |
| --- | --- | --- | --- | --- | --- | --- | --- | --- | --- | --- |
|  | **Cluster 2** | **Cluster 3** | **Cluster 4** | **Cluster 5** | **Cluster 6** | **Cluster 2** | **Cluster 3** | **Cluster 4** | **Cluster 5** | **Cluster 6** |
| **Cluster 1** | ≥.99 | ≥.99 | 0.50 | ≥.99 | *<*.001 | .01 | .11 | *<*.001 | *<*.001 | *<*.001 |
| **Cluster 2** | - | ≥.99 | ≥.99 | ≥.99 | *<*.001 | - | *<*.001 | *<*.001 | 0.36 | *<*.001 |
| **Cluster 3** | - | - | ≥.99 | 0.31 | *<*.001 | - | - | *<*.001 | *<*.001 | .001 |
| **Cluster 4** | - | - | - | 0.08 | *<*.001 | - | - | - | *<*.001 | ≥.99 |
| **Cluster 5** | - | - | - | - | *<*.001 | - | - | - | - | *<*.001 |
|  | **Telehealth, during-pandemic** | | | | | **Office, during-pandemic** | | | | |
|  | **Cluster 2** | **Cluster 3** | **Cluster 4** | **Cluster 5** | **Cluster 6** | **Cluster 2** | **Cluster 3** | **Cluster 4** | **Cluster 5** | **Cluster 6** |
| **Cluster 1** | .002 | *<*.001 | *<*.001 | ≥.99 | ≥.99 | 0.10 | 0.07 | *<*.001 | *<*.001 | *<*.001 |
| **Cluster 2** | - | *<*.001 | *<*.001 | 0.02 | 0.001 | ≥.99 | *<*.001 | *<*.001 | 0.14 | *<*.001 |
| **Cluster 3** | - | - | ≥.99 | *<*.001 | *<*.001 | *<*.001 | ≥.99 | *<*.001 | *<*.001 | *<*.001 |
| **Cluster 4** | - | - | - | *<*.001 | *<*.001 | - | - | - | *<*.001 | *<*.001 |
| **Cluster 5** | - | - | - | - | ≥.99 | - | - | - | - | *<*.001 |

**Table S2.** Dunn’s test results for Psychiatry - appointment durations.

|  | **Telehealth, post-pandemic** | | | | | **Office, post-pandemic** | | | | |
| --- | --- | --- | --- | --- | --- | --- | --- | --- | --- | --- |
|  | **Cluster 2** | **Cluster 3** | **Cluster 4** | **Cluster 5** | **Cluster 6** | **Cluster 2** | **Cluster 3** | **Cluster 4** | **Cluster 5** | **Cluster 6** |
| **Cluster 1** | .002 | ≥.99 | *<*.001 | ≥.99 | *<*.001 | ≥.99 | *<*.001 | *<*.001 | ≥.99 | *<*.001 |
| **Cluster 2** | - | *<*.001 | *<*.001 | *<*.001 | *<*.001 | - | 0.001 | *<*.001 | ≥.99 | *<*.001 |
| **Cluster 3** | - | - | *<*.001 | ≥.99 | *<*.001 | - | - | *<*.001 | *<*.001 | *<*.001 |
| **Cluster 4** | - | - | - | *<*.001 | 0.40 | - | - | - | *<*.001 | ≥.99 |
| **Cluster 5** | - | - | - | - | *<*.001 | - | - | - | - | *<*.001 |
|  | **Telehealth, post-pandemic** | | | | | **Office, post-pandemic** | | | | |
|  | **Cluster 2** | **Cluster 3** | **Cluster 4** | **Cluster 5** | **Cluster 6** | **Cluster 2** | **Cluster 3** | **Cluster 4** | **Cluster 5** | **Cluster 6** |
| **Cluster 1** | *<*.001 | 0.74 | *<*.001 | ≥.99 | 0.41 | ≥.99 | *<*.001 | *<*.001 | ≥.99 | *<*.001 |
| **Cluster 2** | - | *<*.001 | *<*.001 | *<*.001 | *<*.001 | - | 0.09 | *<*.001 | ≥.99 | *<*.001 |
| **Cluster 3** | - | - | *<*.001 | ≥.99 | ≥.99 | - | - | *<*.001 | *<*.001 | *<*.001 |
| **Cluster 4** | - | - | - | *<*.001 | *<*.001 | - | - | - | *<*.001 | 0.02 |
| **Cluster 5** | - | - | - | - | ≥.99 | - | - | - | - | *<*.001 |

**Table S3.** Dunn’s test results for Behavioral Health - patient-to-provider ratios.

|  | **Telehealth, during-pandemic** | | | | | **Office, during-pandemic** | | | | |
| --- | --- | --- | --- | --- | --- | --- | --- | --- | --- | --- |
|  | **Cluster 2** | **Cluster 3** | **Cluster 4** | **Cluster 5** | **Cluster 6** | **Cluster 2** | **Cluster 3** | **Cluster 4** | **Cluster 5** | **Cluster 6** |
| **Cluster 1** | *<*.001 | 0.005 | 0.80 | ≥.99 | ≥.99 | *<*.001 | .55 | *<*.001 | *<*.001 | ≥.99 |
| **Cluster 2** | - | *<*.001 | 0.47 | *<*.001 | ≥.99 | - | *<*.001 | ≥.99 | *<*.001 | 0.03 |
| **Cluster 3** | - | - | *<*.001 | 0.006 | 0.002 | - | - | *<*.001 | *<*.001 | 0.33 |
| **Cluster 4** | - | - | - | ≥.99 | ≥.99 | - | - | - | *<*.001 | *<*.001 |
| **Cluster 5** | - | - | - | - | ≥.99 | - | - | - | - | *<*.001 |
|  | **Telehealth, during-pandemic** | | | | | **Office, during-pandemic** | | | | |
|  | **Cluster 2** | **Cluster 3** | **Cluster 4** | **Cluster 5** | **Cluster 6** | **Cluster 2** | **Cluster 3** | **Cluster 4** | **Cluster 5** | **Cluster 6** |
| **Cluster 1** | *<*.001 | *<*.001 | 0.05 | ≥.99 | 0.14 | *<*.001 | ≥.99 | *<*.001 | *<*.001 | 0.42 |
| **Cluster 2** | - | *<*.001 | ≥.99 | 0.001 | ≥.99 | - | 0.19 | ≥.99 | *<*.001 | ≥.99 |
| **Cluster 3** | - | - | *<*.001 | *<*.001 | *<*.001 | - | - | 0.06 | *<*.001 | ≥.99 |
| **Cluster 4** | - | - | - | 0.11 | ≥.99 | - | - | - | *<*.001 | ≥.99 |
| **Cluster 5** | - | - | - | - | 0.26 | - | - | - | - | *<*.001 |

**Table S4.** Dunn’s test results for Behavioral Health - appointment durations.

|  | **Telehealth, during-pandemic** | | | |  | **Office, during-pandemic** | | | |  |
| --- | --- | --- | --- | --- | --- | --- | --- | --- | --- | --- |
|  | **Cluster 2** | **Cluster 3** | **Cluster 4** | **Cluster 5** | **Cluster 6** | **Cluster 2** | **Cluster 3** | **Cluster 4** | **Cluster 5** | **Cluster 6** |
| **Cluster 1** | ≥.99 | 0.06 | *<*.001 | *<*.001 | ≥.99 | ≥.99 | *<*.001 | *<*.001 | *<*.001 | ≥.99 |
| **Cluster 2** | - | 0.24 | *<*.001 | *<*.001 | ≥.99 | - | *<*.001 | 0.02 | *<*.001 | ≥.99 |
| **Cluster 3** | - | - | *<*.001 | *<*.001 | 0.03 | - | - | *<*.001 | *<*.001 | *<*.001 |
| **Cluster 4** | - | - | - | ≥.99 | *<*.001 | - | - | - | ≥.99 | ≥.99 |
| **Cluster 5** | - | - | - | - | *<*.001 | - | - | - | - | 0.11 |
|  | **Telehealth visits post-pandemic** | | | |  | **Office visits post-pandemic** | | | |  |
|  | **Cluster 2** | **Cluster 3** | **Cluster 4** | **Cluster 5** | **Cluster 6** | **Cluster 2** | **Cluster 3** | **Cluster 4** | **Cluster 5** | **Cluster 6** |
| **Cluster 1** | 0.004 | 0.004 | *<*.001 | *<*.001 | ≥.99 | ≥.99 | *<*.001 | 0.01 | ≥.99 | ≥.99 |
| **Cluster 2** | - | ≥.99 | *<*.001 | *<*.001 | 0.003 | - | *<*.001 | 0.002 | ≥.99 | ≥.99 |
| **Cluster 3** | - | - | *<*.001 | *<*.001 | 0.003 | - | - | *<*.001 | *<*.001 | *<*.001 |
| **Cluster 4** | - | - | - | ≥.99 | 0.003 | - | - | - | 0.17 | 0.74 |
| **Cluster 5** | - | - | - | - | 0.004 | - | - | - | - | ≥.99 |

**Table S5.** Dunn’s test results for Bariatrics - patient-to-provider ratios.

|  | **Telehealth, during-pandemic** | | | |  | **Office, during-pandemic** | | | |  |
| --- | --- | --- | --- | --- | --- | --- | --- | --- | --- | --- |
|  | **Cluster 2** | **Cluster 3** | **Cluster 4** | **Cluster 5** | **Cluster 6** | **Cluster 2** | **Cluster 3** | **Cluster 4** | **Cluster 5** | **Cluster 6** |
| **Cluster 1** | 0.05 | ≥.99 | *<*.001 | 0.01 | *<*.001 | *<*.001 | ≥.99 | *<*.001 | ≥.99 | 0.24 |
| **Cluster 2** | - | 0.09 | *<*.001 | *<*.001 | *<*.001 | - | *<*.001 | 0.1 | *<*.001 | *<*.001 |
| **Cluster 3** | - | - | *<*.001 | *<*.001 | *<*.001 | - | - | *<*.001 | ≥.99 | 0.15 |
| **Cluster 4** | - | - | - | *<*.001 | ≥.99 | - | - | - | *<*.001 | *<*.001 |
| **Cluster 5** | - | - | - | - | *<*.001 | - | - | - | - | ≥.99 |
|  | **Telehealth visits post-pandemic** | | | |  | **Office visits post-pandemic** | | | |  |
|  | **Cluster 2** | **Cluster 3** | **Cluster 4** | **Cluster 5** | **Cluster 6** | **Cluster 2** | **Cluster 3** | **Cluster 4** | **Cluster 5** | **Cluster 6** |
| **Cluster 1** | *<*.001 | *<*.001 | ≥.99 | 0.18 | 0.002 | *<*.001 | ≥.99 | *<*.001 | ≥.99 | 0.04 |
| **Cluster 2** | - | *<*.001 | *<*.001 | *<*.001 | *<*.001 | - | *<*.001 | 0.001 | *<*.001 | *<*.001 |
| **Cluster 3** | - | - | *<*.001 | *<*.001 | *<*.001 | - | - | *<*.001 | ≥.99 | 0.006 |
| **Cluster 4** | - | - | - | 0.02 | 0.01 | - | - | - | *<*.001 | *<*.001 |
| **Cluster 5** | - | - | - | - | *<*.001 | - | - | - | - | 0.04 |

**Table S6.** Dunn’s test results for Bariatrics - appointment durations.

|  | **Telehealth visits post-pandemic** | | | | | **Office visits post-pandemic** | | | | |
| --- | --- | --- | --- | --- | --- | --- | --- | --- | --- | --- |
|  | **Cluster 2** | **Cluster 3** | **Cluster 4** | **Cluster 5** | **Cluster 6** | **Cluster 2** | **Cluster 3** | **Cluster 4** | **Cluster 5** | **Cluster 6** |
| **Cluster 1** | *<*.001 | *<*.001 | 0.002 | 0.03 | ≥.99 | *<*.001 | *<*.001 | 0.030 | *<*.001 | 0.19 |
| **Cluster 2** | - | ≥.99 | ≥.99 | *<*.001 | *<*.001 | - | 0.004 | ≥.99 | ≥.99 | *<*.001 |
| **Cluster 3** | - | - | ≥.99 | 0.007 | *<*.001 | - | - | *<*.001 | *<*.001 | *<*.001 |
| **Cluster 4** | - | - | - | ≥.99 | 0.001 | - | - | - | ≥.99 | *<*.001 |
| **Cluster 5** | - | - | - | - | 0.008 | - | - | - | - | *<*.001 |
|  | **Telehealth visits post-pandemic** | | | | | **Office visits post-pandemic** | | | | |
|  | **Cluster 2** | **Cluster 3** | **Cluster 4** | **Cluster 5** | **Cluster 6** | **Cluster 2** | **Cluster 3** | **Cluster 4** | **Cluster 5** | **Cluster 6** |
| **Cluster 1** | .60 | ≥.99 | 0.10 | ≥.99 | ≥.99 | 0.03 | *<*.001 | ≥.99 | *<*.001 | *<*.001 |
| **Cluster 2** | - | 0.002 | *<*.001 | ≥.99 | ≥.99 | - | *<*.001 | *<*.001 | 0.85 | *<*.001 |
| **Cluster 3** | - | - | 0.68 | 0.07 | ≥.99 | - | - | *<*.001 | *<*.001 | *<*.001 |
| **Cluster 4** | - | - | - | *<*.001 | 0.009 | - | - | - | *<*.001 | *<*.001 |
| **Cluster 5** | - | - | - | - | ≥.99 | - | - | - | - | *<*.001 |

**Table S7.** Dunn’s test results for Sleep Medicine - patient-to-provider ratios.

|  | **Telehealth visits post-pandemic** | | | | | **Office visits post-pandemic** | | | | |
| --- | --- | --- | --- | --- | --- | --- | --- | --- | --- | --- |
|  | **Cluster 2** | **Cluster 3** | **Cluster 4** | **Cluster 5** | **Cluster 6** | **Cluster 2** | **Cluster 3** | **Cluster 4** | **Cluster 5** | **Cluster 6** |
| **Cluster 1** | *<*.001 | *<*.001 | *<*.001 | *<*.001 | 0.01 | ≥.99 | ≥.99 | 0.92 | 0.26 | *<*.001 |
| **Cluster 2** | - | 0.34 | ≥.99 | ≥.99 | *<*.001 | - | 0.04 | *<*.001 | *<*.001 | *<*.001 |
| **Cluster 3** | - | - | 0.91 | 0.25 | *<*.001 | - | - | ≥.99 | ≥.99 | *<*.001 |
| **Cluster 4** | - | - | - | ≥.99 | *<*.001 | - | - | - | ≥.99 | *<*.001 |
| **Cluster 5** | - | - | - | - | *<*.001 | - | - | - | - | *<*.001 |
|  | **Telehealth visits post-pandemic** | | | | | **Office visits post-pandemic** | | | | |
|  | **Cluster 2** | **Cluster 3** | **Cluster 4** | **Cluster 5** | **Cluster 6** | **Cluster 2** | **Cluster 3** | **Cluster 4** | **Cluster 5** | **Cluster 6** |
| **Cluster 1** | ≥.99 | 0.010 | ≥.99 | ≥.99 | *<*.001 | ≥.99 | ≥.99 | *<*.001 | 0.02 | 0.38 |
| **Cluster 2** | - | 0.003 | 0.21 | 0.07 | *<*.001 | - | 0.08 | *<*.001 | *<*.001 | 0.18 |
| **Cluster 3** | - | - | *<*.001 | *<*.001 | *<*.001 | - | - | 0.06 | 0.88 | *<*.001 |
| **Cluster 4** | - | - | - | ≥.99 | *<*.001 | - | - | - | ≥.99 | *<*.001 |
| **Cluster 5** | - | - | - | - | *<*.001 | - | - | - | - | *<*.001 |

**Table S8.** Dunn’s test results for Sleep Medicine - appointment durations.

|  | **Telehealth visits post-pandemic** | | | | | **Office visits post-pandemic** | | | | |
| --- | --- | --- | --- | --- | --- | --- | --- | --- | --- | --- |
|  | **Cluster 2** | **Cluster 3** | **Cluster 4** | **Cluster 5** | **Cluster 6** | **Cluster 2** | **Cluster 3** | **Cluster 4** | **Cluster 5** | **Cluster 6** |
| **Cluster 1** | *<*.001 | 0.45 | *<*.001 | 0.002 | *<*.001 | *<*.001 | 0.003 | *<*.001 | *<*.001 | *<*.001 |
| **Cluster 2** | - | 0.20 | ≥.99 | ≥.99 | 0.006 | - | ≥.99 | *<*.001 | ≥.99 | *<*.001 |
| **Cluster 3** | - | - | 0.002 | ≥.99 | *<*.001 | - | - | *<*.001 | ≥.99 | *<*.001 |
| **Cluster 4** | - | - | - | 0.89 | ≥.99 | - | - | - | *<*.001 | 0.25 |
| **Cluster 5** | - | - | - | - | 0.005 | - | - | - | - | 0.001 |
|  | **Telehealth visits post-pandemic** | | | | | **Office visits post-pandemic** | | | | |
|  | **Cluster 2** | **Cluster 3** | **Cluster 4** | **Cluster 5** | **Cluster 6** | **Cluster 2** | **Cluster 3** | **Cluster 4** | **Cluster 5** | **Cluster 6** |
| **Cluster 1** | *<*.001 | *<*.001 | *<*.001 | *<*.001 | *<*.001 | 0.42 | ≥.99 | *<*.001 | ≥.99 | *<*.001 |
| **Cluster 2** | - | ≥.99 | *<*.001 | *<*.001 | ≥.99 | - | ≥.99 | *<*.001 | ≥.99 | *<*.001 |
| **Cluster 3** | - | - | *<*.001 | 0.003 | 0.23 | - | - | *<*.001 | ≥.99 | *<*.001 |
| **Cluster 4** | - | - | - | *<*.001 | *<*.001 | - | - | - | *<*.001 | *<*.001 |
| **Cluster 5** | - | - | - | - | *<*.001 | - | - | - | - | *<*.001 |
