# Supplementary material for: Resource Use Patterns in US Telehealth Services: Machine Learning and Clustering Analysis Across 4 Specialties
Source: JMIR Med Inform. 2026 May 7;14:e78030. doi: 10.2196/78030 (PMC13195373; doi:10.2196/78030)
Supplement: Multimedia Appendix 9 [file medinform_v14i1e78030_app9.docx]

To provide a comprehensive view of the U.S., we calculate county-level RUCA codes by aggregating census tract-level RUCA codes, weighted by each tract’s population (Figure S1). RUCA codes range from 1 to 10, each representing varying levels of urbanization and commuting patterns. Figure S2 illustrates broadband subscription rates across RUCA codes, showing a clear trend: broadband subscription rates generally decline as RUCA codes increase. This suggests that more rural areas (with higher RUCA codes) face greater challenges in broadband access, underscoring the importance of considering broadband availability and affordability when analyzing rural-urban disparities.


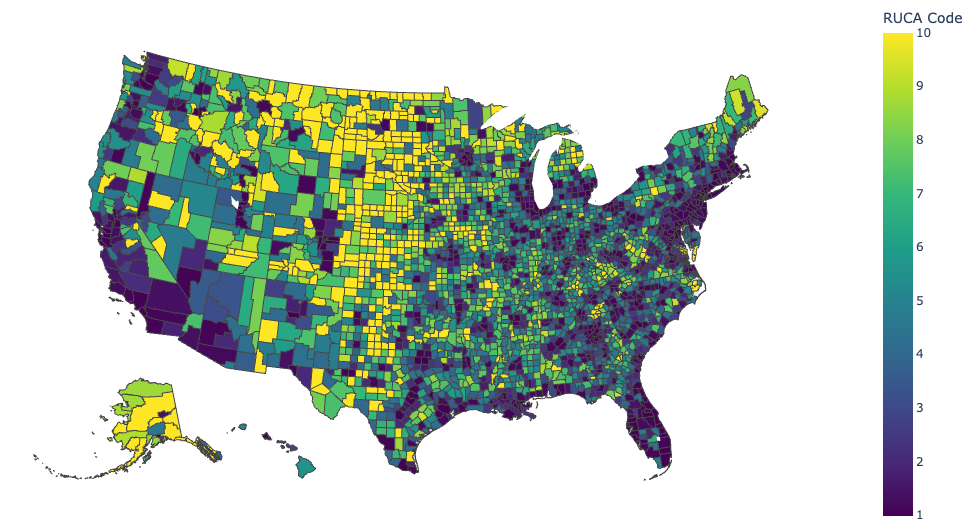


**Figure S1.** RUCA codes of US counties.


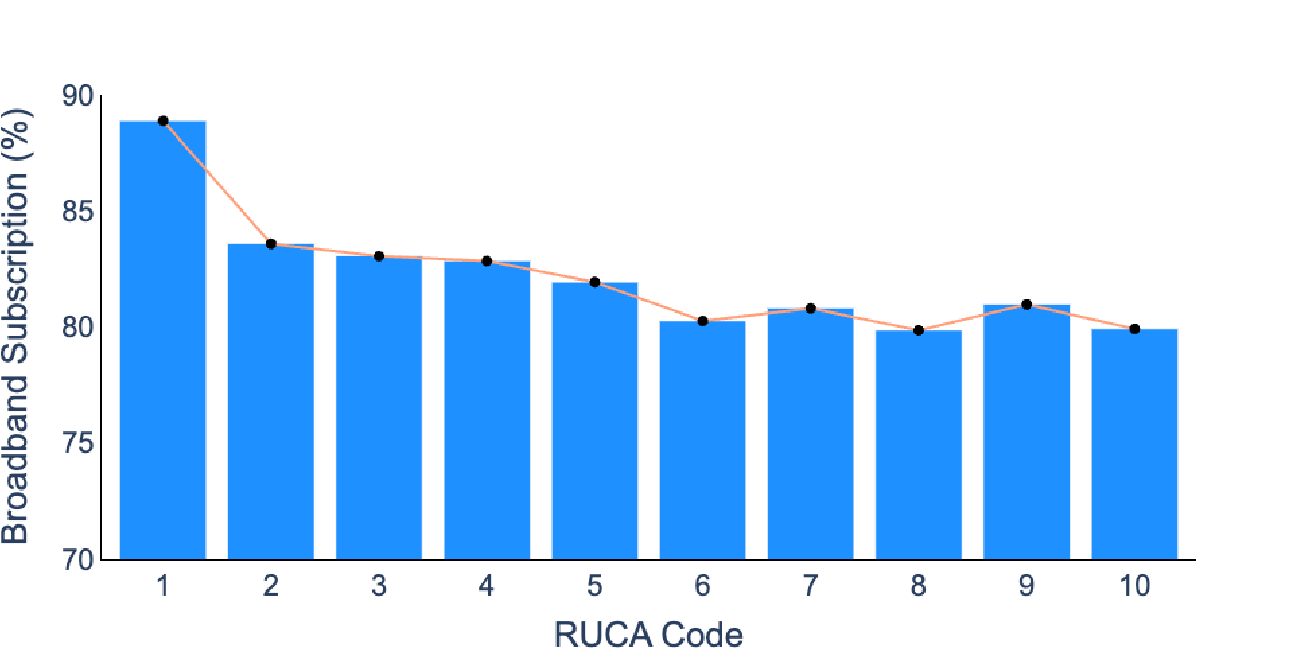


**Figure S2.** Broadband subscriptions by RUCA codes.
